# Nonspecific binding of common anti-CFTR antibodies in ciliated cells of human airway epithelium

**DOI:** 10.1038/s41598-021-02420-x

**Published:** 2021-12-01

**Authors:** Yukiko Sato, Kamila R. Mustafina, Yishan Luo, Carolina Martini, David Y. Thomas, Paul W. Wiseman, John W. Hanrahan

**Affiliations:** 1 Department of Physiology, McGill University, McIntyre Medical Sciences Building, 3655 Promenade Sir William Osler, Montréal, QC H3G 1Y6 Canada; 2 Cystic Fibrosis Translational Research Centre, McGill University, Montréal, Canada; 3 Department of Chemistry, McGill University, Montréal, Canada; 4 Department of Biochemistry, McGill University, Montréal, Canada; 5 Department of Physics, McGill University, Montréal, Canada; 6 Research Institute – McGill University Health Centre, Montréal, Canada

**Keywords:** Cellular imaging, Mechanisms of disease, Membrane trafficking, Cell biology, Physiology, Molecular medicine, Pathogenesis

## Abstract

There is evidence that the cystic fibrosis transmembrane conductance regulator (CFTR) anion channel is highly expressed at the apical pole of ciliated cells in human bronchial epithelium (HBE), however recent studies have detected little CFTR mRNA in those cells. To understand this discrepancy we immunostained well differentiated primary HBE cells using CFTR antibodies. We confirmed apical immunofluorescence in ciliated cells and quantified the covariance of the fluorescence signals and that of an antibody against the ciliary marker centrin-2 using image cross-correlation spectroscopy (ICCS). Super-resolution stimulated emission depletion (STED) imaging localized the immunofluorescence in distinct clusters at the bases of the cilia. However, similar apical fluorescence was observed when the monoclonal CFTR antibodies 596, 528 and 769 were used to immunostain ciliated cells expressing F508del-CFTR, or cells lacking CFTR due to a Class I mutation. A BLAST search using the CFTR epitope identified a similar amino acid sequence in the ciliary protein rootletin X1. Its expression level correlated with the intensity of immunostaining by CFTR antibodies and it was detected by 596 antibody after transfection into CFBE cells. These results may explain the high apparent expression of CFTR in ciliated cells and reports of anomalous apical immunofluorescence in well differentiated cells that express F508del-CFTR.

## Introduction

Human airway bronchial epithelium contains ciliated cells, secretory goblet and club cells, basal cells, and pulmonary neuroendocrine cells^1,2^, each with distinct roles in airway physiology and host defense. Cystic fibrosis transmembrane conductance regulator (CFTR) is a cAMP regulated anion channel required for normal secretion of airway surface liquid^3–5^. CFTR mutations lead to abnormal mucus and impaired mucociliary clearance of inhaled bacteria that are hallmarks of cystic fibrosis (CF)^6^.

CFTR is thought to be highly expressed in ciliated cells of the airway epithelium, however recent single cell mRNA sequencing (scRNAseq) reports detected low CFTR mRNA levels in ciliated cells from primary human bronchial epithelial (HBE) cultures and mouse lung tissue^7–9^. It was suggested that most CFTR transcripts (> 45%) are in a rare (< 2% of total) epithelial cell type called pulmonary ionocytes^7,8^, although one scRNAseq study found most CFTR transcripts (80% of the total) in secretory and basal cells^9^. Further analysis at the protein level is needed to assess CFTR immunofluorescence in ciliated cells and understand F508del-CFTR apical immunofluorescence reported in some studies of CF airways^10,11^.

Many CFTR mutations have been identified and classified as Class I—VI based on the predominant molecular defects they produce^12–14^. Class I mutations include nonsense, frameshift and splicing mutations that prevent expression of the full-length protein. Class II mutations cause protein misfolding and impair trafficking to the plasma membrane. Class III mutations are those that inhibit channel gating or regulation, while class IV–VI mutations reduce pore conduction, protein expression and CFTR stability, respectively. F508del is by far the most frequent mutation, occurring on at least one chromosome in ~ 90% of the CF population. It is a Class II mutation that causes misfolding, retention in the endoplasmic reticulum, and premature degradation by the proteasome and other pathways^15–17^.

We used monoclonal antibodies to immunofluorescence label and localize CFTR in well-differentiated primary cultures of human bronchial epithelial cells from non-CF donors and F508del homozygotes. Importantly, we also immunostained well-differentiated cells from CF patients homozygous for rare Class I mutations that cause CFTR truncation upstream of the epitope for three antibodies. CFTR and the ciliary marker protein centrin-2 immunofluorescence signals were localized by confocal microscopy, and spatial image cross-correlation spectroscopy (ICCS) was used to quantify their co-localization and biomolecule interaction fractions^18,19^. Finally, we characterized CFTR immunostaining using confocal and super-resolution stimulated emission depletion (STED) microscopy^20^. Although all antibodies detected CFTR with high sensitivity in undifferentiated and transfected cells, several also recognized another protein in ciliated cells from a subset of cell donors and the cross-reacting antigen was investigated.

## Materials and methods

### Cell culture

Primary human bronchial epithelial cells (HBEs) were obtained from the Primary Airway Cell BioBank at McGill University (https://mcgill.ca/cftrc/platforms/primary-airway-cell-biobank-pacb). Cells were isolated from CF patients (F508del/F508del or Class I/Class I) undergoing lung transplantation. CF lung tissues were from the Respiratory tissue Biobank at the Centre hospitalier de l'Université de Montréal Research Centre (CRCHUM) and were obtained with informed consent following protocols approved by the Institutional Review Boards at the CRCHUM and McGill University (#A08-M70-14B). Non-CF lungs were obtained from the National Development and Research Institutes, Inc. (NDRI, New York, NY) and International Institute for the Advancement of Medicine (IIAM, Edison NJ). First passage cells were seeded on collagen coated polyester membrane inserts (Corning) and maintained in air–liquid interface (ALI) media under submerged conditions for 3–4 days. The apical medium was then removed to promote differentiation for ≥ 21 days^21^. Parental CFBE41o^-^ cells lines were kindly provided by Dieter Gruenert (UCSF, San Francisco, CA) and cultured in EMEM containing 10% FBS, 5% Pen/Strep, 5% L-glutamine. Baby hamster kidney (BHK) cells stably expressing WT-CFTR were cultured in Dulbecco's Modified Eagle Medium/Nutrient Mixture F-12 (DMEM/F12) containing 10% FBS, 5% Pen/Strep and 500 µM methotrexate. pEGFP Rootletin (Nigg pFL2(CW499), Addgene plasmid # 41,166; http://n2t.net/addgene:41166)^22^ and eGFP-rootletinX1 (Genscript) were transfected using Genejuice according to the manufacturer’s instructions.

### Immunofluorescence imaging and analysis

pHBE cells were transduced using mCherry-WTCFTR adenovirus in ALI medium without BSA and Pen/Strep. After 16 h infection the medium was replaced with complete ALI medium and cells were differentiated at the ALI for 10–15 days. To immunostain well-differentiated pHBE cells, they were washed with PBS and either fixed immediately or gently scraped and centrifuged for 5 min onto coverslips at 450 rpm using a Cytospin 4 (Thermo Fisher). They were then fixed with 4% PFA (Thermo Fisher) for 15 min, permeabilized using 0.5% Triton X-100 (Sigma-Aldrich) for 15 min and blocked with 2% BSA (Sigma-Aldrich) for 45 min. Immunostaining was performed with the following mouse monoclonal anti-CFTR antibodies: 596 (CFFT, 1:200), 528 (CFFT, 1:200), 769 (CFFT, 1:200), 450 (CFFT, 1:200), 217 (CFFT, 1:200), mouse-anti-CFTR570 (CFFT, 1:200), MM13-4 (Millipore Sigma, 1:200). The following antibodies were also used: mouse anti-tubulin (Millipore, 1:200), rabbit anti-muc5A (Santa Cruz, 1:200), rabbit anti-cytokeratin 14 (PTGlab, 1:200) and mouse anti-rootletin (Santa Cruz, 1:200). Primary antibody was added for 16 h at 4 °C, then goat anti-mouse Alexa Fluor 488 (Thermo Fisher, 1:1000) or goat anti-rabbit Alexa Fluor 596 (Thermo Fisher 1:1000). Nuclei were stained using DAPI (Sigma, 0.5 μg/ml). The cells were mounted in Prolong Gold mounting medium and imaged using a confocal LSM 780 (Zeiss). Images were collected using Zen Software and processed using ImageJ. The average background intensity was subtracted and the brightness and contrast were adjusted and kept constant between all conditions. To test blocking by CFTR-and rootletin-based peptides (WPSGGQMT and WSPGGQML(Genscript), respectively) they were preincubated with mAb596 antibody on an orbital shaker before immunostaining as described above. Fixed samples were preincubated in antigen retrieval buffer (Tris, 5% urea, pH 9.5, 95 °C) for 20 min prior to rootletin immunostaining to ensure access to the epitope.

### STED microscopy

After performing immunostaining of differentiated pHBE cells on intact supports with the primary antibodies, secondary immunostaining was performed for 1 h at 22 °C using anti-mouse STAR 635P (Abberior, 1:1000) and anti-rabbit STAR 580 (Abberior, 1:1000) antibodies, followed by mounting with Abberior Mount Solid (Abberior) or 2,2'-thiodiethanol (Sigma-Aldrich). STED imaging was performed at 100x (NA 1.4) magnification using an Abberior Instruments Quad Scanning STED microscope with 594 and 640 nm excitation lasers and a 1.5 W, 775 nm depletion laser. Data was acquired using Imspector software (Abberior Instruments).

### Image cross-correlation spectroscopy (ICCS)

Spatial image cross-correlation spectroscopy was used to extract the information about the spatial organization of the labeled proteins in the membrane^23^. The fluctuations in fluorescence intensity in channel $a$ can be defined as:

1 $$\begin{array}{*{20}c} {\delta I_{a}\left( {x, y} \right) = I_{a} \left( {x,y} \right) - \left\langle I \right\rangle _{a} } \\ \end{array}$$

where $I _{a} \left( {x,y} \right)$ and $\left\langle I \right\rangle _{a}$ are the intensity at pixel position $\left( {x,y} \right)$ and the spatial average intensity of the pixels in the selected ROI, respectively. Furthermore, the number of interacting particles in the beam focal spot $\left\langle N_{a} \right\rangle$ can be calculated as the inverse of the ratio between the mean square fluctuation to the average intensity of fluorescence in the channel ROI:

2 $$\begin{array}{*{20}l} \langle{N_{a}\rangle^{ - 1} = \frac{{\left\langle\left( {\delta I _{a} } \right\rangle\right)^{2} }}{{ \left\langle I_{a} \right\rangle ^{2} }} } \\ \end{array}$$

Direct calculation of accurate particle densities is usually not possible due to the presence of noise in the intensity measurements. However, a white noise-free estimate can be calculated from a correlation function calculation and fit to obtain the zero-lags amplitude of a normalized spatial intensity fluctuation correlation function. If $\xi$ and $\eta$ are spatial lags (pixel shifts) in channels $a$ and $b$, respectively, then spatial cross-correlation or autocorrelation ($a = b$) functions can be calculated with Eq. (3):

3 $$\begin{array}{*{20}l} {r\left( {\xi ,\eta } \right)_{{ab}} = \frac{{\left\langle {\delta I_{a} \left( {x,y} \right)\delta I_{b} \left( {x + \xi ,y + \eta } \right)} \right\rangle }}{{\left\langle I \right\rangle _{a} \left\langle I \right\rangle _{b} }}} \hfill \\ \end{array}$$

Evaluating Eq. (3) at 0 spatial lags yields Eq. (2) and hence the inverse of the particle density.

To estimate $r\left( {0,0} \right)_{ab}$, or the zero-lags amplitude of correlation functions, Eq. (3) can be fitted as the two-dimensional Gaussian function of the following form:

4 $$\begin{array}{*{20}l} {r\left( {\xi , \eta } \right)_{ab} = r\left( {0,0} \right)_{ab} \exp \left[ { - \frac{{\left( {\xi - u} \right)^{2} + \left( {\eta - v} \right)^{2} }}{{\omega_{0}^{2} }}} \right] + r_{\infty } } \\ \end{array}$$

where $$\omega_{0}$$ is the $$e^{ - 2} $$ beam radius, $$\left( {u,v} \right)$$ is the position of the peak maximum, and $$r_{\infty }$$ is the offset parameter to account for the long-range spatial correlations.

For the imaging channels $a$ and $b$, the autocorrelation function can be calculated for each channel, followed by the spatial cross-correlation function. Two-dimensional Gaussian (Eq. 4) fitting is then used to obtain the best estimation for the correlation function zero-lags amplitudes $r\left( {0,0} \right)_{aa}$, $r\left( {0,0} \right)_{bb}$ and $r\left( {0,0} \right)_{ab}$. The colocalization coefficients can be calculated as the ratios between the number of interacting particles to the total number of particles, via the ratio of correlation function amplitudes, in channel $a$ as $M1$ and channel b as $M2$ ^24^:

5 $$\begin{array}{*{20}l} {M1_{{{\text{ICCS}}}} = \frac{{r\left( {0,0} \right)_{ab} }}{{r\left( {0,0} \right)_{bb} }} = \frac{{ \left\langle N \right\rangle _{{ab}} }}{{ \left\langle N \right\rangle _{{aa}} }}} \\ \end{array}$$

6 $$\begin{array}{*{20}l} {M2_{{{\text{ICCS}}}} = \frac{{r\left( {0,0} \right)_{ab} }}{{r\left( {0,0} \right)_{aa} }} = \frac{{ \left\langle N \right\rangle _{{ab}} }}{{ \left\langle N \right\rangle _{{bb}} }} } \\ \end{array}$$

Analysis was performed on 64 × 64 pixels representative ROIs of cell apical domains. Custom MATLAB ICCS code is available on Wiseman Lab GitHub (https://github.com/Wiseman-Lab/ICCS-CFTR).

### RNA extraction and qPCR

RNA was isolated using the Illustra™ RNAspin mini kit (GE healthcare life science) and reverse transcribed using SuperScript VILO Mastermix (Thermo Fisher) for 1 h at 42 °C, and for 5 min at 85 °C. The cDNA (250 ng), 10 μl TaqMan® Fast Advanced Mastermix, 1 μl TaqMan® Gene Expression Assay primers (GAPDH-Hs02786624_g1, CFTR-Hs00357011_m1) in a total volume of 20 μl were placed in the wells of a MicroAmp EnduraPlate™ Optical 96-Well Fast Reaction Plate. qPCR was performed using a QuantStudio™ 7 Flex Real-Time PCR system with the following protocol: 20 s at 95 °C and 40 cycles at 95 °C (1 s) and 60 °C (20 s) and this was followed by ΔΔCT analysis.

### Short-circuit current

Cells were mounted in modified Ussing chambers (Physiological Instruments, San Diego, CA). The basolateral solution contained (mM): 115 NaCl, 25 NaHCO_3_, 1.2 MgCl_2_, 1.2 CaCl_2_, 2.4 KH_2_PO_4_, 1.24 K_2_HPO_4_ and 10 D-glucose). The apical solution contained (mM): 1.2 NaCl, 115 Na-gluconate, 25 NaHCO_3_, 1.2 MgCl_2_, 4 CaCl_2_, 2.4 KH_2_PO_4_, 1.24 K_2_HPO_4_ and 10 D-glucose). The monolayer was clamped to 0 mV and currents were recorded using a Powerlab 8SP A-D converter as described previously^25^.

### Immunoblotting

Cells were washed, then lysed in RIPA buffer (0.15 M NaCl, 20 mM Tris–HCl pH 8.0, 0.08% sodium deoxycholate, 1% Triton X-100 (Sigma-Aldrich), 0.1% SDS and protease inhibitor cocktail (Roche)). Lysate protein (10 µg for BHK-WT, 40 µg for pHBE cells) was resolved using SDS-PAGE and transferred to nitrocellulose membranes. The membranes were blocked using 5% (w/v) skim milk powder in TBS (20 mM Tris–HCl at pH 7.4, 150 mM NaCl) for 1 h at 22 °C, then incubated with mouse-anti-CFTR (23C5, mAb developed by our group, 1:200), mouse-anti-CFTR 596 (CFFT, 1:1000), mouse-anti-CFTR 528 (CFFT, 1:1000), mouse-anti-CFTR 769 (CFFT, 1:1000), mouse-anti-CFTR 450 (CFFT, 1:1000), mouse-anti-CFTR 217 (CFFT, 1:1000), mouse-anti-CFTR 570 (CFFT, 1:1000), mouse anti-CFTR MM13-4 (Millipore Sigma, 1:1000), anti-tubulin (Sigma, 1:1000) or anti-rootletin (Santa Cruz, 1:1000) for 16 h at 4 °C. Then the membrane was washed 4 × with TBST for 15 min at 22 °C, incubated with secondary antibody (goat-anti-mouse-HRP, Jackson, 1:1000) for 1 h at 22 °C, and washed 4 × with TBST. The bands were visualized using Pierce™ ECL Western Blotting Substrate (Thermo Fisher) and a ChemiDoc Imaging system (BioRad).

### Cilia measurements and proliferation assays

Well differentiated pHBE cells were washed with PBS, gently scraped and centrifuged onto coverslips at 450 rpm for 5 min using a Cytospin 4 (Thermo Fisher). Cells were then fixed, and the length of the cilia was measured in bright field images using the Zen software. The value obtained for each sample of ciliated cells was an average of 3–5 measurements^26^. Proliferation was quantified by seeding 5 × 10^4^ HBE cells in 24 well plates that had been coated with PureCol collagen in BEGM medium. At the indicated time points, cells were rinsed with PBS, trypsinized, and counted using a hemocytometer.

### Statistical analysis

Data were analyzed by one-way ANOVA. P < 0.05 was considered significant and analyses were performed using Prism 5 for Mac OS X (GraphPad Software, CA).

## Results

### CFTR immunofluorescence in transfected cells

**Figure 1:**
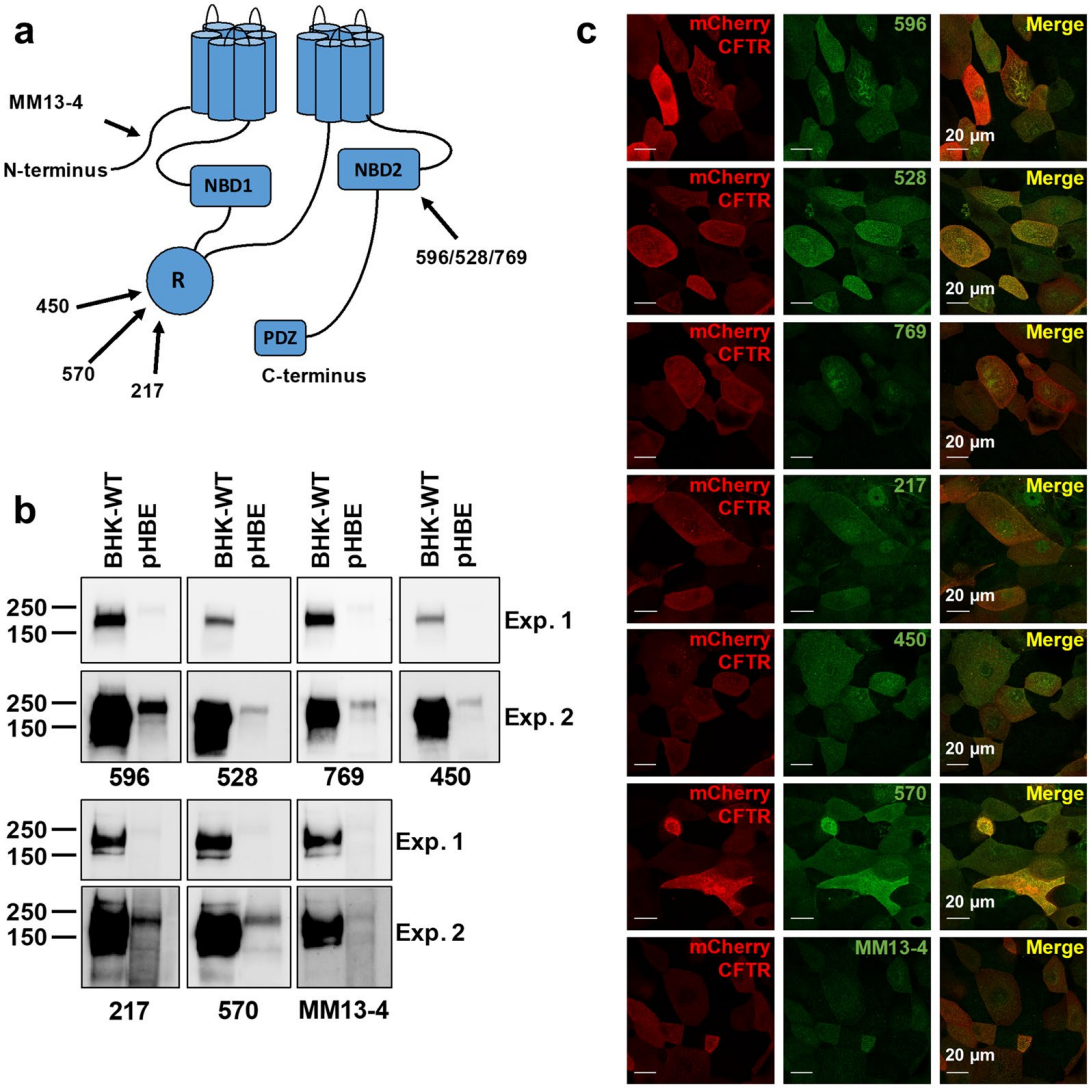
Immunodetection of CFTR. (**a**) Cartoon showing locations of epitopes for the seven anti-CFTR antibodies used in this study. (**b**) Immunoblots of lysates after SDS-PAGE probed for CFTR using each antibody. BHK-WT cells were stably transfected with CFTR. Well differentiated primary bronchial epithelial (HBE) cells expressed endogenous CFTR. Protein (BHK-10 µg, HBE-40 µg) was detected after short and long exposures (Exp.1 and 2, respectively). Blots were cropped. (**c**) Confocal images of HBEs at the apical membrane after transduction with adenoviral mCherry-WTCFTR. Cells were cultured at the ALI for 10–15 days and immunostained on intact membrane support.

We began by studying the monoclonal antibodies 596, 528 and 769, which recognize a common epitope in NBD2 and are often used to immunostain CFTR. For comparison we studied antibodies 450, 570 and 217, which recognize non-overlapping linear epitopes in the R-domain, and MM13-4, which is directed against the N-terminus (Fig. 1a)^10,27–32^. To determine antibody sensitivity and specificity we compared blots of stably transfected BHK cell lysates (for heterologous CFTR) and well-differentiated HBE cells (for detection of endogenous CFTR) using short and long chemiluminescence exposures (Exp. 1 and 2, respectively). All seven antibodies recognized both immature (band B) and complex glycosylated (band C) glycoforms of CFTR in BHK cells (Fig. 1b). They also detected CFTR in HBE cell lysates, which had much lower expression as expected. The band C glycoform was most abundant in HBE cells (Fig. 1b). These results confirmed the utility of the antibodies for immunoblotting under these conditions^30^.

To test the sensitivity and specificity of immunostaining, CF HBE cells were transduced with mCherry-WTCFTR and differentiated at the air–liquid interface (ALI) for 10–15 days. All CFTR antibodies tested detected heterologous CFTR and their fluorescence signals overlapped with that of mCherry (Fig. 1c, Supplementary Fig. S1). Antibodies 217, 528, MM13-4 also caused diffuse immunofluorescence in untransduced cells, which may reflect low endogenous CFTR expression or non-specific background staining (Fig. 1c*,* Supplementary Fig. S1). In summary, all seven antibodies recognize heterologous CFTR in BHK cells and endogenous CFTR in HBE cells by immunoblotting, and yield strong immunofluorescence that appears specific in HBE cells transduced with mCherry-WTCFTR.

### Antibodies 596, 528, 769 and 217 immunostain the bases of motile cilia

**Figure 2:**
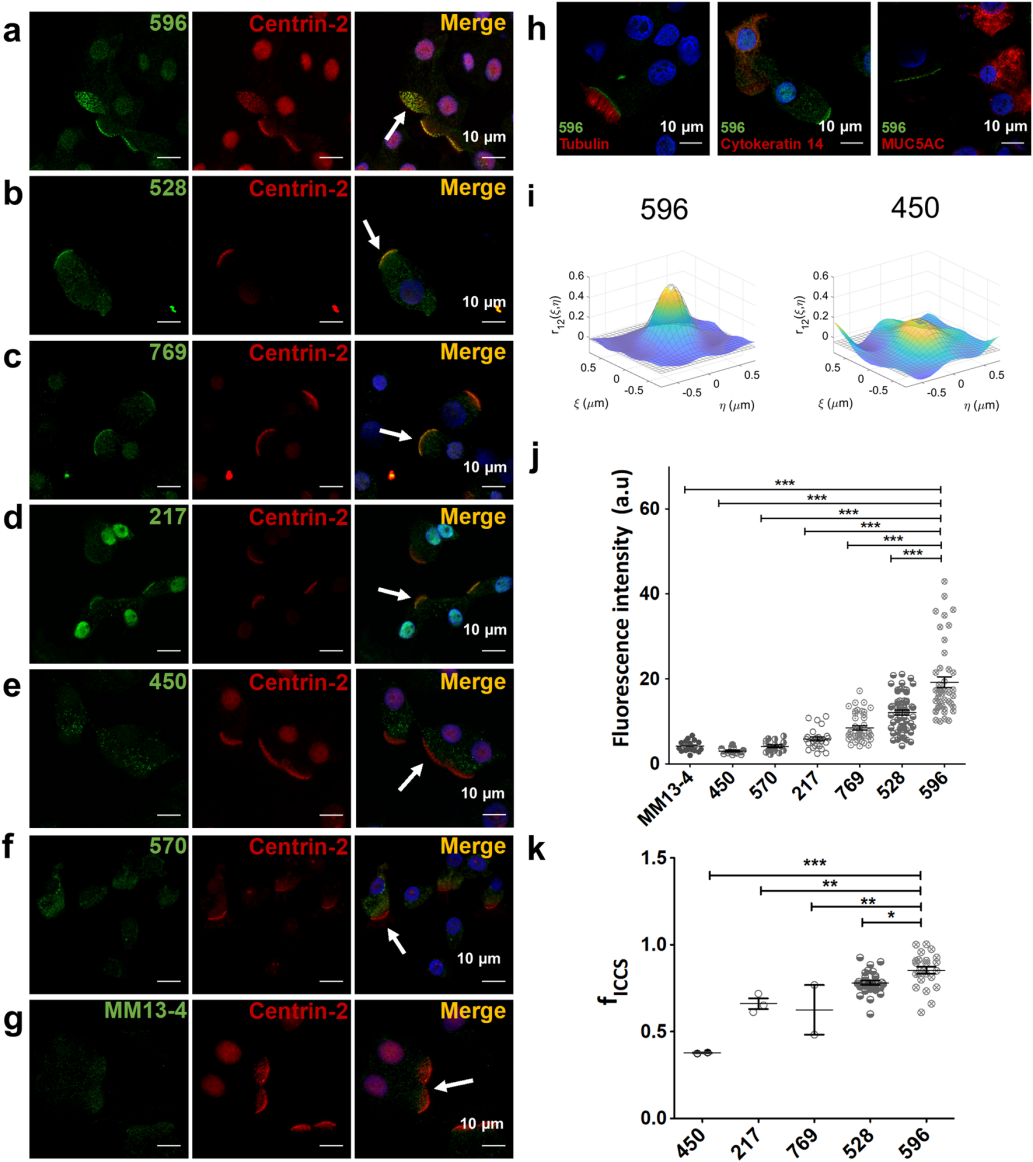
The bases of cilia are immunostained using CFTR antibodies 596, 528, 769 and 217, but not 450, 570 and MM13-4. (**a–g**) Confocal images of well differentiated HBEs co-immunostained with CFTR antibodies 596, 528, 769, 217, 450, 570 or MM13-4 and the ciliated cell marker centrin-2**.** Arrows indicate ciliated cells. (**h**) Confocal images of cells coimmunostained with 596 and antibodies against either tubulin (left, ciliated cell marker), cytokeratin 14 (middle, basal cell marker) or MUC5AC (right, goblet cell marker). (**i**) Representative spatial cross-correlation functions calculated via ICCS analysis between centrin-2 and CFTR immunofluorescence using 596 (left) or 450 (right) measured from image sub-regions of interest (64 × 64 pixels). (*j*) Average apical immunofluorescence signals with different CFTR antibodies. Intensities were normalized to the excitation power (mean ± SD, n = 12–57, ***p < 0.0001, one-way ANOVA). (**k**) Colocalized fraction f_ICCS_ extracted from ICCS analysis (mean ± SD, n = 2–28, 450 vs 596, ***p < 0.0001; 217 vs 596, **p = 0.005; 769 vs 596, **p = 0.0059; 528 vs 596, *p = 0.0313), which indicates the arithmetic mean of the fractions of CFTR and centrin-2 antibody signals that interact.

We then examined ciliated cells, which were identified by co-immunostaining the ciliary marker proteins centrin-2 (basal body) or acetylated tubulin (axoneme). Antibodies 596, 528, 769 and 217 yielded robust fluorescence signals at the apical pole of ciliated cells as indicated by the white arrows in the merged images (Fig. 2a–d, j) consistent with previous reports^10,29,30^. The signals were strongest using 596, 528 and 769 and they were clearly situated at the bases of cilia (Fig. 2a–c). Similar results were obtained when 596 was used with HBE cultures from six non-CF donors expressing wild-type CFTR (Supplementary Fig. S2). However, WT-CFTR immunofluorescence was not observed at the apical pole of ciliated cells using the monoclonal antibodies 450, 570 and MM13-4 (Fig. 2e–g) or in basal and goblet cells using 528, 769, 217 or 596 (Fig. 2h, Supplementary Fig. S3). The 596 immunofluorescence in ciliated cells was intense, comparable to pulmonary ionocytes identified using an antibody against the ionocyte marker FOXi1 (Supplementary Fig. S3d), suggesting ciliated cells and ionocytes have similar levels of CFTR.

**Figure 3:**
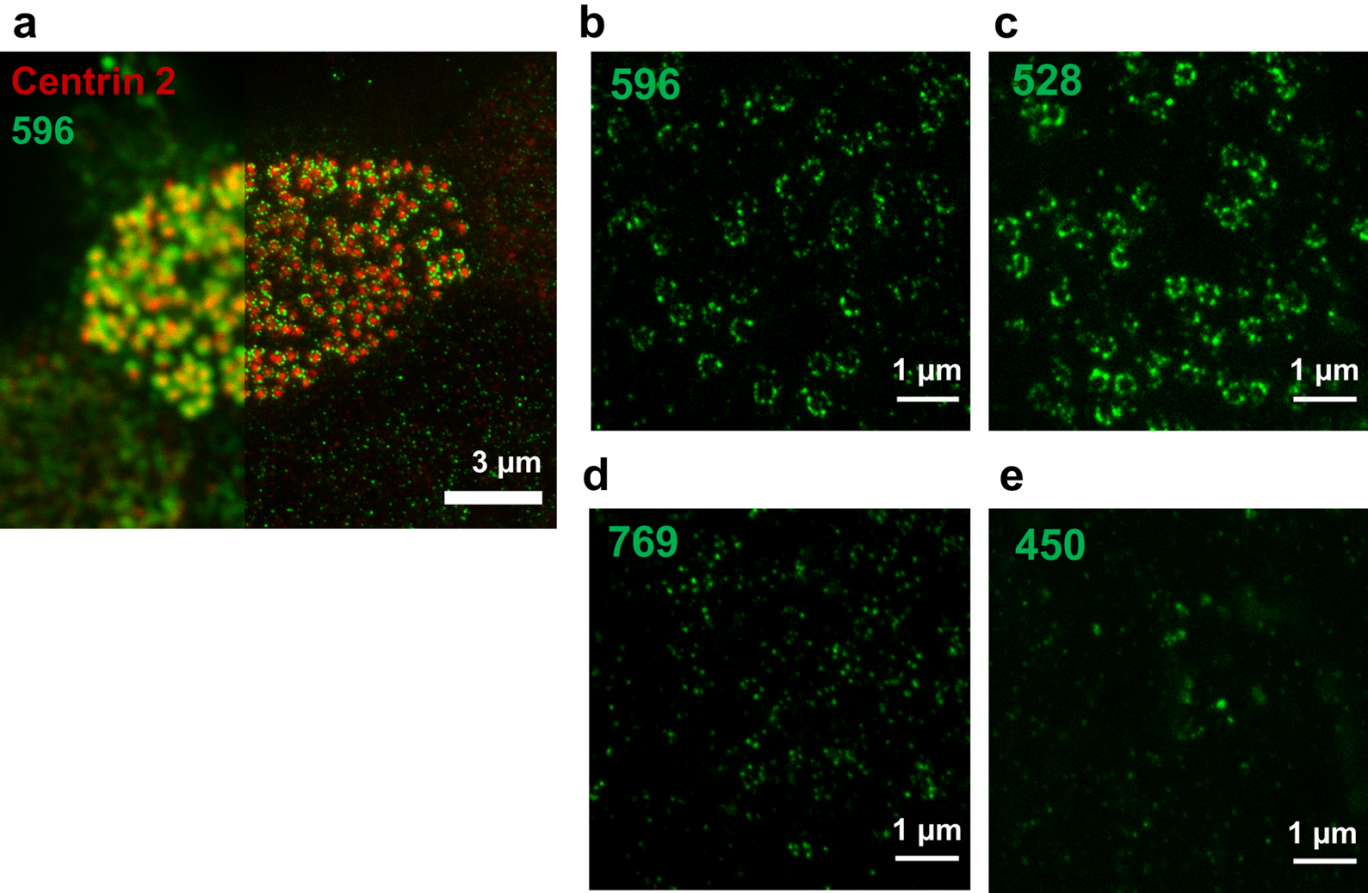
Super-resolution microscopy of 596 immunofluorescence reveals ring-like pattern at the bases of cilia. (**a**) Representative image of the apical side of a non-CF HBE cell co-immunostained with 596 and centrin-2 antibody; left side: confocal image, right side: super-resolution STED image. (**b–e**) Representative STED images of apical membrane fluorescence in HBE cells obtained using 596, 528, 796 and 450 antibodies.

To study apical immunofluorescence in ciliated cells quantitatively we determined the colocalization fraction of fluorescence signals produced by antibodies against CFTR and the ciliary marker centrin-2 using spatial image cross-correlation spectroscopy (ICCS) analysis. Cross-correlation functions were calculated from the fluorescence intensity fluctuations in the two image channels and colocalization was determined by fitting them with 2D Gaussian distributions (Fig. 2i, Supplementary Fig. S4). The beam focal spot areas and amplitudes of the fitted auto- and cross-correlation functions were used to calculate the interaction parameters M1 and M2, where M1 is the fraction of anti-CFTR antibody fluorescence (green) colocalizing with anti-centrin-2 antibody fluorescence (red), and M2 is the colocalization fraction of anti-centrin-2 antibody with anti-CFTR antibody. We report the colocalized fraction f_iccs_ as the arithmetic mean of M1 and M2 parameters^33^. The signals that were most highly colocalized with centrin-2 were those of 596 (f_iccs_ = 0.85 ± 0.10) and 528 (f_iccs_ = 0.78 ± 0.07), followed by 217 (f_iccs_ = 0.66 ± 0.04) and 769 (f_iccs_ = 0.63 ± 0.14) (Fig. 2k, Supplementary Fig. S4). By contrast, the interacting fraction for 450 was low (f_iccs_ = 0.377 ± 0.002), while fits using fluorescence signals obtained with 570 and M13-4 did not meet the signal-to-noise criteria in the Methods, precluding correlation analyses. These results indicate that anti-CFTR antibodies can be distinguished based on their colocalization with centrin-2. The discrepancies in colocalization are larger than the predicted distance between epitopes on CFTR (~ 4 nm), further evidence that the CFTR antibodies recognize different proteins. We examined ciliary immunofluorescence at higher spatial resolution (~ 50 nm) using super-resolution (STED) imaging. The 596 and centrin-2 signals appeared as discrete clusters, with the 596 immunofluorescence clusters in a circle around centrin-2 (Fig. 3a). A similar pattern was observed using the NBD2 antibodies 528 and 769 (Fig. 3b–d), but not with the R domain antibody 450 (Fig. 3e). These results indicate that 217, 596, 528 and 769 immunostain the apical pole of ciliated cells and that antibodies that detect an epitope in NBD2 (596, 528 and 769) detect a ring of clusters around the base of each motile cilium.

### Antibodies 596, 528 and 769 stain ciliated cells from most, but not all, F508del homozygotes

**Figure 4:**
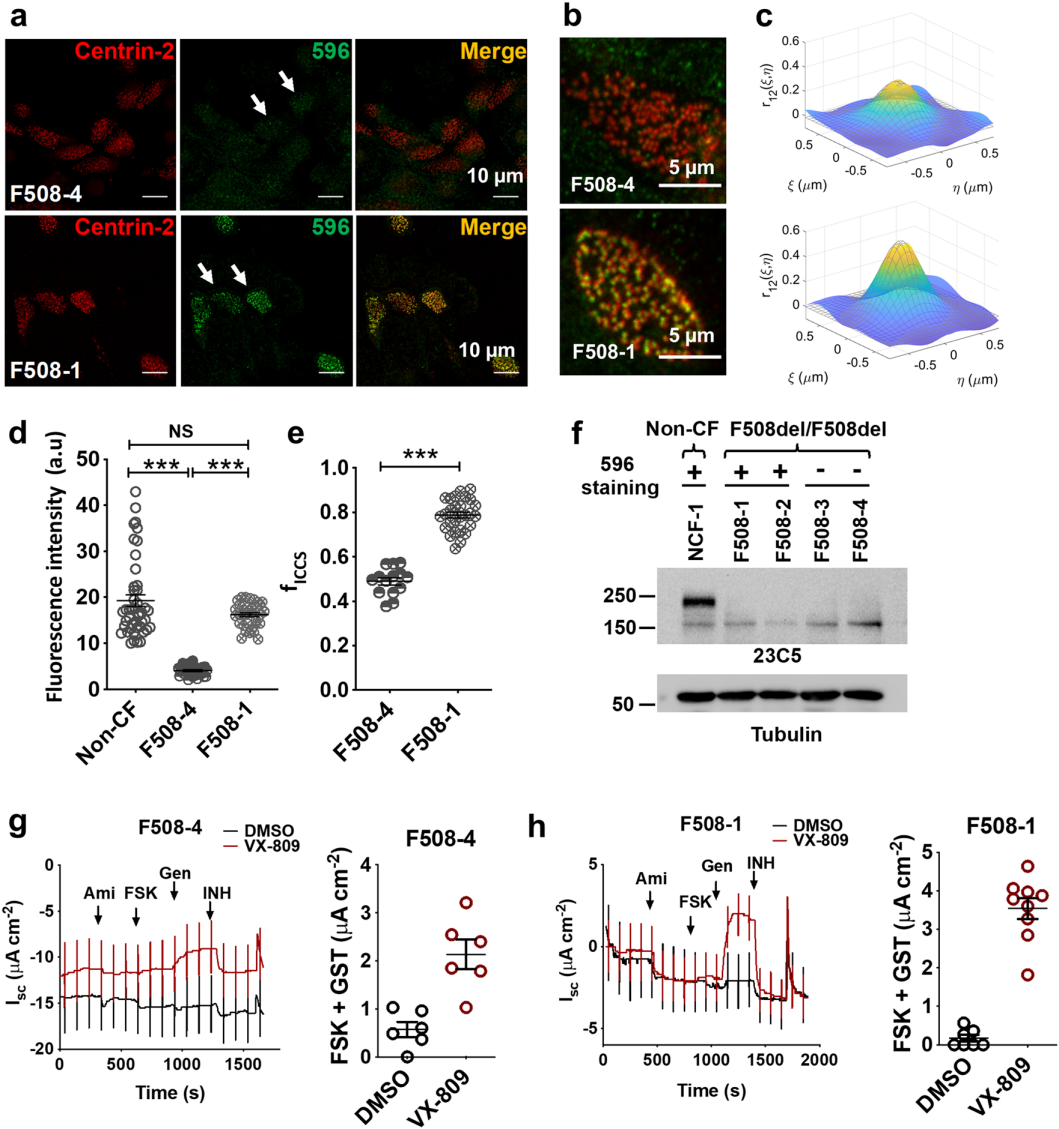
Apical 596 immunofluorescence in pHBE cells isolated from a subset of F508del/F508del patients. (**a**) Confocal images of apical immunofluorescence using well differentiated HBE cells from two F508del/F508del patients, one 596- (F508-4, top row) and the other 596+ (F508-1, bottom row). Colocalization with centrin-2 immunofluorescence confirmed expression in ciliated cells (merge). (**b**) Confocal images of the apical surface of F508-4 (top row) and F508-1 (bottom row) immunostained with 596 (green) and centrin-2 (red) antibodies used for image cross-correlation spectroscopy analysis. (**c**) Examples of spatial cross-correlation functions calculated using ICCS analysis from sub-regions (64 × 64 pixels) of the ciliated region images used to determine colocalization values; i.e. fractions of interacting particles per beam area in the green channel (M1) and the red channel (M2), for F508-4 (top) and F508-1 (bottom). (**d**) Summary of apical 596 immunofluorescence intensities in F508-4 and F508-1 cells. Intensity was normalized to the excitation power, (mean ± SD, n = 28—34, ***p < 0.0001, one-way ANOVA). (**e**) Colocalized fraction f_ICCS_ extracted from ICCS analysis (mean ± SD, n = 14–26, ***p = 6 × 10^−16^, t-test). (**f**) Immunoblot of lysates from well differentiated F508del/F508del HBE cells probed with antibody 23C5 against the R domain of CFTR. (**g**, **h**, these images are from one blot). Short-circuit current recordings and summaries of maximal cAMP responses after 24 h pretreatment with vehicle (DMSO, black) or the corrector VX-809 (1 µM, red). Cells were exposed sequentially to amiloride (Ami; 100 µM), forskolin (FSK; 10 µM) and genistein (Gen; 50 µM). Currents were inhibited using CFTR_inh_-172 (INH; 10 µM). Note that under control conditions (DMSO), F508-1 cells with apical 596 immunofluorescence had smaller stimulated currents compared to F508-4 cells that were 596-.

The F508del mutation causes misfolding and premature degradation of CFTR so that little is present on the plasma membrane of transfected cells^15^. Nevertheless, apical CFTR immunofluorescence has been reported in primary airway epithelial cells expressing F508del CFTR^10,30,31^. When well-differentiated CF HBE cells were immunostained using seven anti-CFTR antibodies, 217 yielded immunofluorescence at the bases of cilia in all CF (F508del/F508del) and non-CF cells examined (Supplementary Fig. S5d). FOXi1+ cells (ionocytes) that have high endogenous CFTR expression were rare under these culture conditions, thus antibodies 450, 570 and MM13-4 did not stain a particular cell type and only a diffuse signal that was similar in non-CF and CF cells was detected (Supplementary Fig. S5e–g). Interestingly, 596, 528 and 769 stained the apical pole of CF cells in a subset of patients (7/10), suggesting variable expression of the cross-reacting protein between individuals (Fig. 4a, Supplementary Figs. S5a–c, S6). Figure 4 and Supplementary Fig. 6 shows representative cells with very low (F508-4 and F508-3) and high (F508-1 and F508-2) apical 596 immunofluorescence intensity, which we refer to henceforth as 596- and 596+ , respectively.

ICCS analysis yielded different colocalization parameters for 596+ and 596- cells (Fig. 4b–e; Supplementary Fig. S7a, b). The fractions of 596 and centrin-2 signals colocalizing in 596+ CF cells (f_iccs_ = 0.79 ± 0.07) were significantly higher than in 596- CF cells (f_iccs_ = 0.49 ± 0.06), suggesting the 596 signal resembles background immunofluorescence (and therefore is less correlated with centrin-2). The results with NBD2 antibodies contrast with those obtained using 450, 570 and MM13-4, which did not stain a specific cell type in F508del HBE cultures and had the same pattern in CF and non-CF ciliated cells as mentioned above (Supplementary Fig. S5e–g). Apical immunofluorescence was fourfold higher in 596 + cells compared to 596- cells when 450, 570 or MM13-4 were used (Fig. 4d) and was comparable to that in non-CF cells expressing WT-CFTR (~ 20 arbitrary units). By contrast, immunostaining by 217 was similar in F508del and non-CF cells suggesting it cross-reacts with a different antigen (Supplementary Fig. S5d). Surprisingly intense apical 596 immunofluorescence in CF cells prompted us to check if there was biosynthetic arrest of F508del CFTR under our culture conditions. However this was confirmed, as only the immature (band B) glycoform of the F508del/F508del was detected by immunoblotting lysates from four CF patients despite robust apical 596 immunofluorescence. Both band B and mature band C wild-type CFTR were detected in non-CF cells as expected (Fig. 4f).

Functional assays provided further evidence that strong apical 596 immunofluorescence was unrelated to CFTR channel activity. Forskolin and genistein caused a negligible increase in short-circuit current across 596+ CF cells and Isc responses were increased by 24 h pretreatment with the CFTR corrector VX-809 (Fig. 4g, h). Exposure to the correctors VX-445 and VX-661 (correctors that are in Trikafta) did not affect apical immunostaining in 596- ciliated cells, but increased electrophysiological responses to forskolin (Supplementary Fig. S8). These results indicate that defects in F508del-CFTR trafficking and channel function are not correlated with apical 596 immunofluorescence in ciliated cells.

### Apical 596 immunofluorescence in ciliated cells that are homozygous for a Class I CFTR mutation

**Figure 5:**
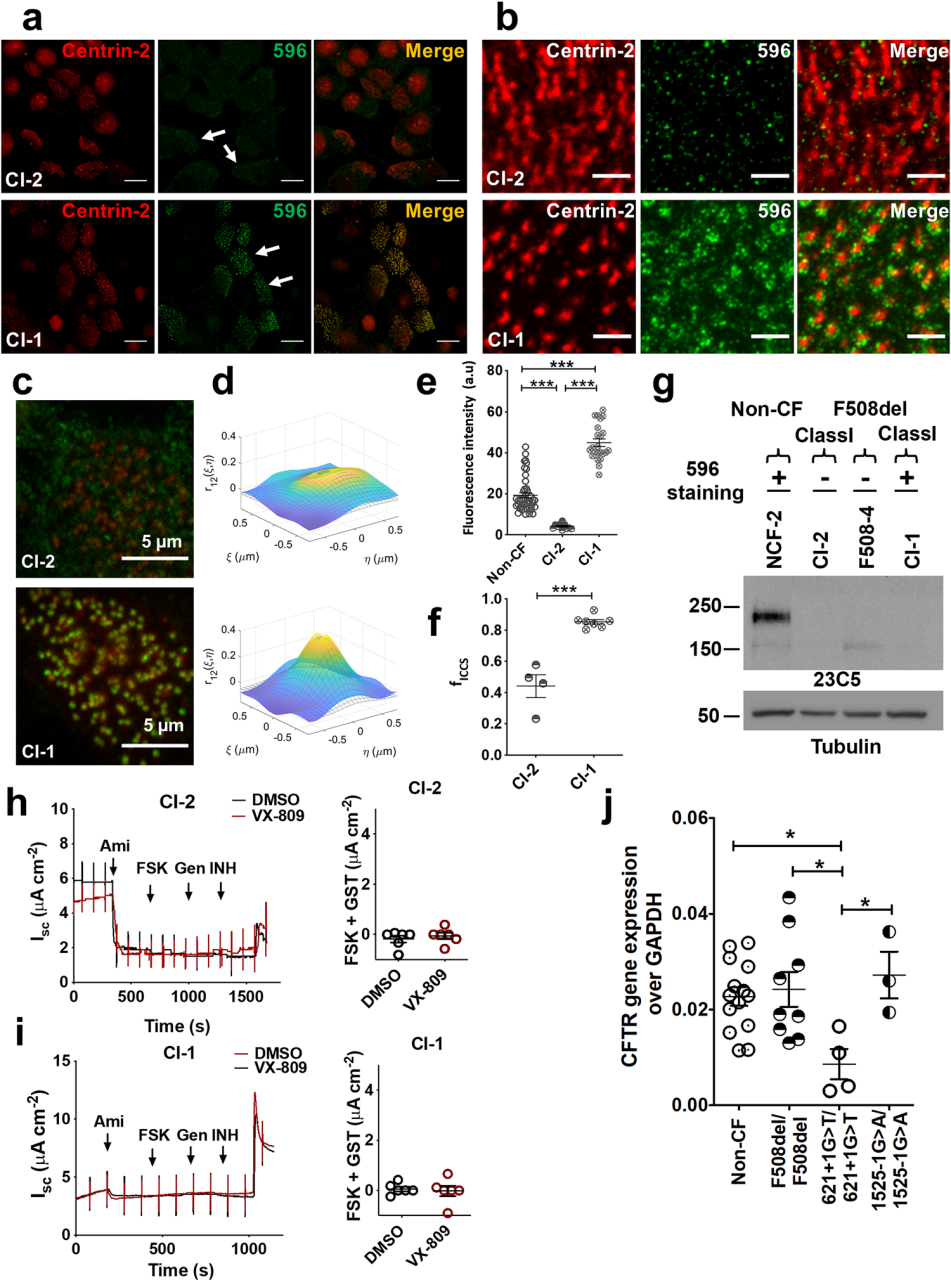
Apical 596 immunofluorescence in ciliated cells that are homozygous for Class I CFTR mutations. (**a**) Confocal images of well differentiated pHBE cells from patients homozygous for 1525-1G > A (CI-2, top row) or 621 + 1G > T (CI-1, bottom row). (**b**) Representative STED images of the apical surface of CI-2 (top row) or CI-1 (bottom row) cells. (**c**) Confocal images of the apical surface of CI-2 (top row) and CI-1 (bottom row) cells immunostained with 596 and centrin-2 antibodies used for ICCS analysis. (**d**) Examples of spatial cross-correlation functions calculated via ICCS analysis from sub-regions (64 × 64 pixels) of the ciliated region images used to calculate colocalization values for CI-2 (top) and CI-1 (bottom)**.** (**e**) Fluorescence intensity of 596 immunostaining at the apical pole of CI-2 and CI-1 cells (mean ± SE, n = 12–23, ***p < 0.0001, one-way ANOVA). Intensity was normalized to the excitation power. (*f*) Colocalized fraction f_ICCS_ extracted from ICCS analysis (mean ± SD, n = 3–6, ***p = 5 × 10^–5^, t-test). (**g**) Immunoblot of well differentiated HBE cells from non-CF, class I mutations, or F508del, probed for CFTR using the mAb23C5. Note that CI-1 cells are immunostained by 596 but do not have detectable CFTR protein (**h**, **I**, these images are from one blot). Representative short-circuit current recordings and after pretreatment with DMSO (black) or VX-809 (1 µM, red) for 24 h. Cells were exposed to amiloride (Ami; 100 µM), forskolin (FSK; 10 µM), genistein (Gen; 50 µM) and CFTR_inh_-172 (INH; 10 µM). The histogram shows stimulations induced by FSK + Gen. (*j*) CFTR mRNA levels in well differentiated HBE cells from non-CF donors and patients homozygous for F508del and a Class I mutation. Data were obtained by qPCR using primers that amplify sequence near the 5’ end of the transcripts and results were normalized to GAPDH (mean ± SE, n = 3–14; non-CF vs 621 + 1G > T/621 + 1G > T, *p = 0.0360; F508del/F508del vs 621 + 1G > T/621 + 1G > T, *p = 0.02727; 1525-1G > A/1525-1G > A vs 621 + 1G > T/621 + 1G > T, *p = 0.0413; one-way ANOVA). Individual points represent independent measurements.

Loss of signal in tissues after gene knock out provides the most widely accepted test of antibody specificity. To validate CFTR antibodies in well differentiated airway epithelium we used HBE cells that are null for full length CFTR due to homozygous Class I *cftr* mutations. We compared apical 596 immunostaining in cells with the *cftr* genotype 621 + 1G > T/621 + 1G > T (cell donor CI-1) and 1525-1G > A/1525-1G > A (cell donor CI-2). These mutations cause defective splicing in introns 4 and 9 that results in premature termination codons in the first transmembrane domain and NBD1, respectively. Both stop codons truncate CFTR upstream of the epitopes for antibodies 596, 528, 769 and 217. Surprisingly, ciliated cells from donor CI-1 (which produce CFTR lacking NBD2) had robust apical 596 immunofluorescence resembling non-CF cells (Fig. 5a, c–e), while CI-2 cells had eightfold lower immunofluorescence under identical conditions (Fig. 5b, c–e).

These results were confirmed and extended using super-resolution microscopy. In cells from donor CI-1, 596 immunofluorescence formed small rings of bright clusters (Supplementary Fig. S9) whereas little 596 immunofluorescence was detected in cell homozygous for the other Class I mutation (CI-2, Fig. 5b, c). ICCS analyses yielded much higher correlation-measured colocalization of 596 with centrin-2 in 596+ CI-1 cells (f_iccs_ = 0.85 ± 0.04) compared to 596- CI-2 cells (f_iccs_ = 0.44 ± 0.15); (Fig. 5c–f; Supplementary Fig. S7c, d). The broad Gaussian fit to the cross-correlation function for CI-2 cells likely reflects the lower total immunofluorescence intensity of 596 in those cells compared to CI-1 (44 AU vs 4 AU) (Fig. 5e). These results indicate ciliated cells lacking CFTR still have apical 596 immunostaining, which varies between different cell donors as observed above for F508del CFTR cells. We confirmed that these Class I mutant cells do not express full length CFTR (Fig. 5g) and do not have forskolin- and genistein-activated short-circuit currents after pretreatment with VX-809 (Fig. 5h, i). PCR analysis also revealed that the 621 + 1G > T homozygote (donor CI-1) had ~ 50% less CFTR mRNA compared to non-CF cells, despite having similar apical 596 immunofluorescence (Fig. 5j).

Class I mutant cells were also used to validate other CFTR antibodies used in this study. The bases of cilia were stained in CI-1 but not CI-2 cultures using 528 and 769, which recognize the same NBD2 epitope as 596 (Supplementary Fig. S10b, c). More surprisingly, the R domain antibody 217 immunostained the bases of cilia in cells homozygous for wild-type CFTR or Class I mutants, suggesting it cross-reacts with a different protein from the one recognized by 596, 528 and 769 (Supplementary Fig. S10d). Apical immunofluorescence in ciliated cells was negligible using 450, 570 or MM13-4 regardless of the genotype (i.e. homozygous for Class I, F508del or wild-type CFTR), suggesting their diffuse intracellular signals are due to nonspecific binding (Supplementary Fig. S10e–g).

In summary, 596, 528 and 769 recognize a protein situated at the bases of cilia in CF cells lacking CFTR and has variable expression between individuals. Antibody 217 apparently detects a different protein at the apical pole of all HBE cells examined. By contrast, the R domains antibodies 450 and 570 appear more specific as they did not immunostain Class I patients or ciliated cells from non-CF donors, consistent with recent scRNAseq studies.

### A blocking peptide abolishes the immunostaining of ciliated cells by 596

**Figure 6:**
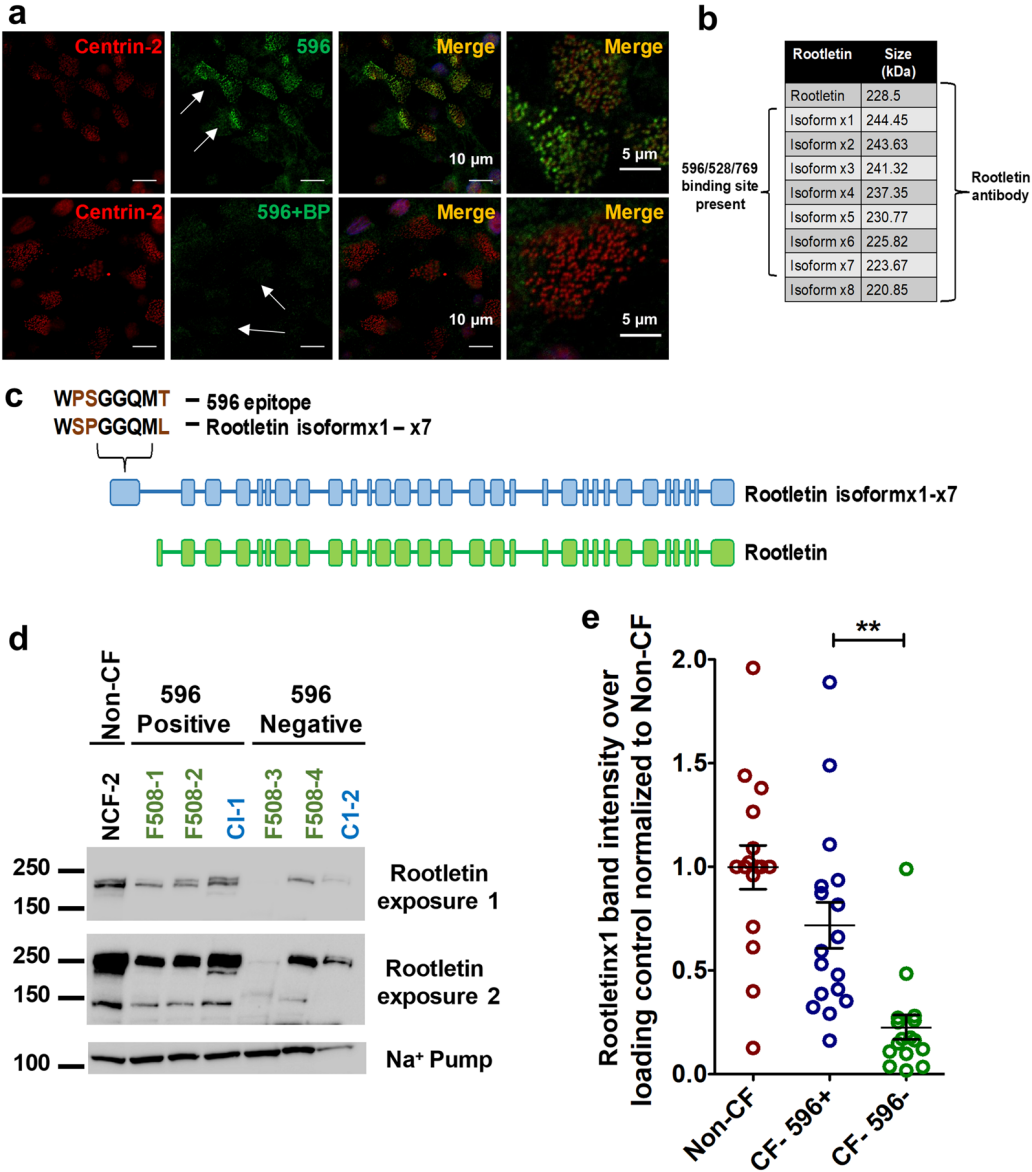
Blocking peptide WPSGGQMT abolishes 596 immunostaining at the bases of cilia. (**a**) Well differentiated HBEs were co-immunostained using 596 and centrin-2 antibodies without (top row) or with (bottom row) a blocking peptide corresponding to the 596 epitope**.** White arrows indicate ciliated cells. Zoomed-in images are shown at extreme right. (**b**) Rootletin isoforms containing a sequence homologous to the 596 epitope in CFTR. (**c**) Cartoon showing location of the pseudo-epitope in rootletin isoforms. (**d**) Immunoblot of well differentiated HBE cell lysates from a non-CF donor and six CF patients probed using rootletin antibody. Green and blue labels indicate donor is homozygous for F508del and a Class I mutation, respectively. Short (1) and long (2) exposures are shown. After short exposure note that rootletin was only detected in 596+ cell lysates. (**e**) Summary of rootletinX1 protein expression. Each point is the lysate from a different culture prepared from five non-CF donors, five 596+ donors and three 596- donors. Note that rootletinX1 band intensity is significantly lower in cells that are not immunostained using CFTR antibody 596 (mean ± SE, n = 16–17, **p = 0.0018, one-way ANOVA).

Variable apical 596 immunostaining in ciliated cells was not correlated with the age or sex of the cell donors (Supplementary Table S2). Antibodies 596, 528 and 769 all recognize the sequence WPSGGQMT on CFTR (Supplementary Table S3) and would be expected to cross-react with the same protein. The ability of these antibodies to detect CFTR on denaturing SDS-PAGE gels (Fig. 1b) implies that amino acid sequence is the main determinant of binding, and this was further suggested by the ability of an 8-mer peptide with this sequence to block immunostaining by 596 (Fig. 6a).

### CFTR antibody 596 recognizes both endogenous and heterologously expressed rootletin

A BLAST search using the NBD2 epitope sequence produced several hits, the most promising of which was rootletinX1-X7 (Supplementary Table S3). Rootletin is a ~ 220 kDa structural protein expressed at the bases of motile cilia^34–36^. It has nine variants, seven of which contain a sequence near the N-terminus that resembles the 596 epitope in CFTR (Fig. 6b, c).

To test if CFTR antibodies detect rootletin variants in well-differentiated HBE cells we began by immunoblotting HBE cell lysates from non-CF donors, and from 596+ and 596- CF cells (i.e. with and without apical immunostaining, respectively). We observed bands consistent with multiple rootletin variants and their densities were lower in cells that were not immunostained by 596 (Fig. 6d, e). This was not due to 596- cultures having fewer ciliated cells as there was no correlation between ciliation area and either the intensity of 596 immunostaining or the density of bands on immunoblots (Supplementary Fig. S11).

**Figure 7:**
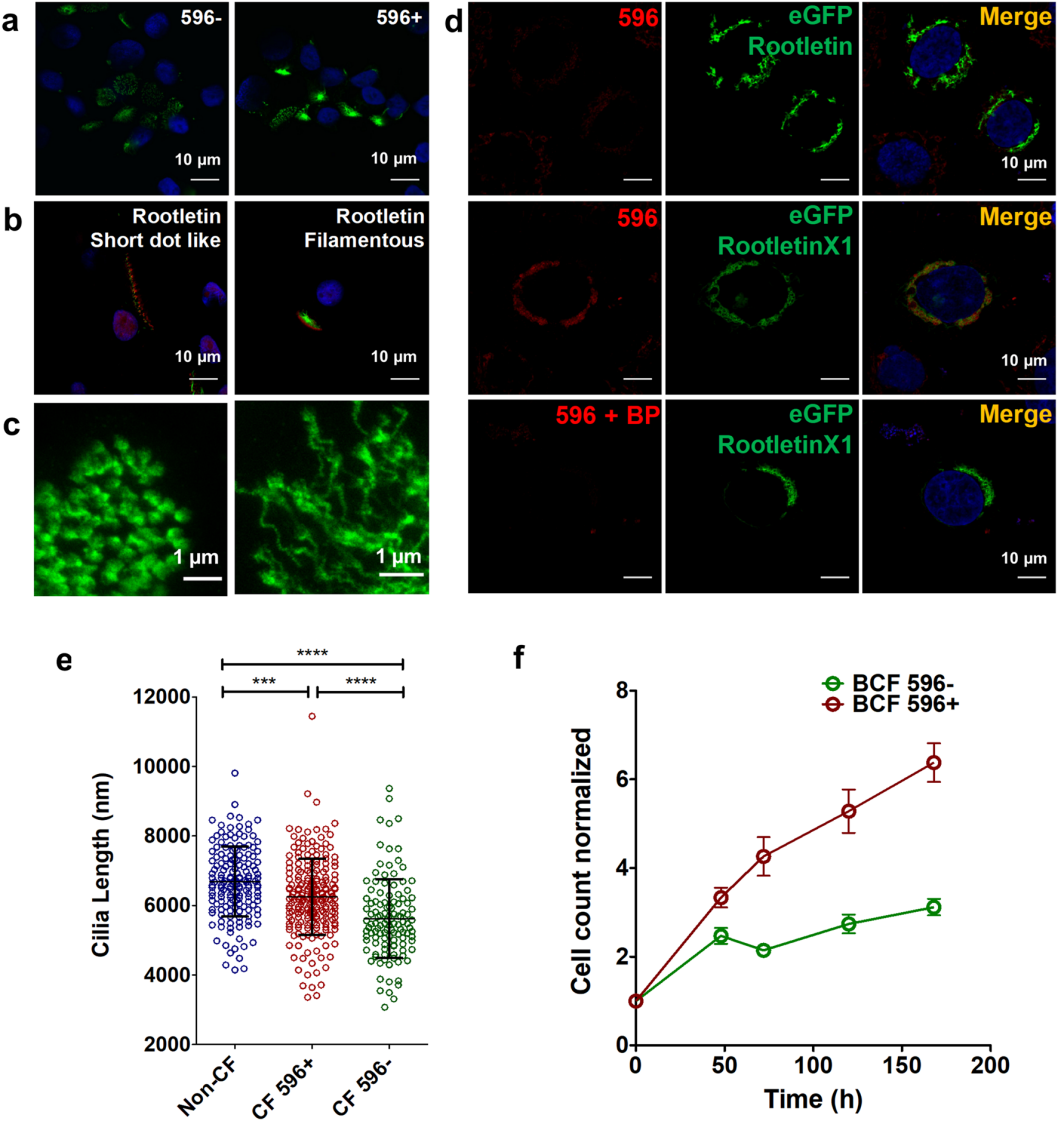
Cross-reactivity of 596 with rootletin and the phenotype of 596- cells. (**a**, **b**) Immunostaining of 596- (left) and 596+ (right) cells with rootletin antibody. (**c**) STED images of 596+ cells showing both punctate and filamentous rootletin immunofluorescence. (**d**) Confocal images of eGFP-tagged rootletin (lacking WSPGGQML, top row) and the isoform rootletinX1 (which has the pseudo-epitope, middle and bottom rows) expressed in CFBE41o^-^ cells. RootletinX1 was detected using 596 antibody (red) and this immunostaining was abolished by blocking peptide (BP). (**e**) Cilia length in non-CF, 596+ and 596- cells (mean ± SE, n = 119–125, ****p < 0.0001, ***p = 0.0004, one-way ANOVA). Each point represents a single cell. (**f**) Comparison of 596+ and 596- proliferation rates. Each point represents the mean cell count ± SE of 3 independent experiments.

Immunostaining with anti-rootletin antibody also yielded lower rootletin immunofluorescence in 596- CF cells compared to 596+ cells (Fig. 7a), consistent with immunoblots probed using anti-rootletin antibody (Supplementary Fig. S11). Confocal and super-resolution imaging of the rootletin antibody signal revealed both filamentous and punctate structures at the bases of the cilia (Fig. 7b, c).

To test if 596 recognizes exogenous rootletin variants we expressed eGFP-rootletin (which lacks the pseudo-epitope WSPGGQML) and eGFP-rootletinX1 (which has this sequence) in CFBE cells and immunostained them using 596, 528 or 769 (Fig. 7d and Supplementary Fig. S12a–c). As shown in Fig. 7d, 596 colocalized with eGFP-rootletinX1 but not with eGFP-rootletin. Adding excess WPSGGQMT peptide (the CFTR epitope sequence) blocked the immunostaining of eGFP-rootletinX1 by 596. Moreover 528 and 769, which bind the same NBD2 epitope on CFTR, also recognized rootletinX1 (Supplementary Fig. S12b, c) whereas 217, 450 and 570 directed against other sequences in NBD2 and the R domain did not (Supplementary Fig.S12d–f). We then tested if a peptide with the pseudo-epitope sequence prevents 596 immunostaining at the bases of cilia. A reduction was observed following prolonged exposure to a blocking peptide (Bp) bearing the Rootletin1X pseudo-epitope sequence WSPGGQML (Supplementary Fig. S13a, b). By contrast, immunostaining by 217 was not reduced by this blocking peptide and was actually increased slightly by exposure to blocking peptide with the 596 epitope sequence WPSGGQMT (Supplementary Fig. S13c*, *d). These results indicate that 596 cross-reacts with rootletin variants bearing the sequence WSPGGQML whereas 217 does not and presumably cross-reacts with a different protein.

### Cells lacking 596 immunofluorescence have shorter cilia and proliferate slowly

To explore the possible significance of variable rootletin expression we measured the length of the cilia in 596- and 596+ cells and its impact on cell proliferation. Cells that were not immunostained by 596 displayed a small, but significant reduction in ciliary length (Fig. 7e), consistent with a previous report that rootletin knockdown mice have increased cilia breakage^37^. Rootletin serves as a centriole linker and could promote mitosis and epithelial repair after injury. Consistent with this role, we found that 596+ HBE cells proliferate more rapidly than 596- cells (Fig. 7f).

## Discussion

This study examined the distribution of CFTR in well differentiated HBE cells. Using several antibodies we observed apical immunofluorescence in non-CF and F508del ciliated cells, and in cells that were effectively null for CFTR due to a Class I mutation. The results demonstrate that several widely used antibodies that share a common epitope on CFTR cross-react with another protein in well differentiated ciliated cells, probably recognizing a rootletin variant such as rootletin X1 found in the basal bodies of motile cilia. We found that rootletin X1 expression varied between individuals, resulting in cross-reactivity in cells from some but not all cell donors. This reconciles reports of strong apical CFTR immunofluorescence and low mRNA transcript levels in ciliated cells and may also explain unexpected apical immunostaining in well differentiated F508del-CFTR airway cells.

Early studies demonstrated immunofluorescence at the apical membrane of ciliated cells in native airway tissue and in primary cultures using the monoclonal antibodies MATG1061 (epitope aa 722–734) and MATG1104 (aa 503–515) from Transgène SA, and the polyclonal antibody PAC865 (aa 1202–1422), however no difference was observed between the signal detected in cells expressing wild-type CFTR and those that were homozygous for F508del^38^. The monoclonal antibody 528 yielded strong immunofluorescence at the apical pole of non-CF ciliated cells which was absent in F508del/F508del cells as expected^29^. Those results could be explained if the CF cells had low rootletinX1 expression. Anomalous apical immunofluorescence has been reported in freshly isolated nasal cells from F508del homozygous patients using the monoclonal antibodies MM13-4 and MATG1061 and the polyclonal antibody pAb169, albeit in a reduced number of cells compared to those from non-CF donors. Further studies are needed to understand apparent trafficking of F508del CFTR in nasal cells^10,30,31^.

Using 596 and several other CFTR antibodies we observed strong apical immunostaining of ciliated cells from non-CF patients, but found similar signals in cells from seven out of ten F508del/F508del patients. We also detected apical immunofluorescence in well-differentiated CF HBE cells that were null for CFTR due to the Class I splicing mutation 621 + 1G > T. These results indicate 596 and related antibodies cross-react with another protein in well differentiated ciliated cells. When apical CFTR immunofluorescence was detected in ciliated cells the intensity was surprisingly strong; i.e., comparable to that in ionocytes identified using FOXi1 as a marker. Thus, cross-reactivity could explain the paradoxical immunostaining of ciliated cells using 596, 769, 528, 217 and low mRNA levels in those cells by single cell RNA sequencing^7–9^. Non-specific binding is not unique to CFTR antibodies. When 9,358 internally generated antibodies and 5,436 commercial antibodies from 51 different vendors were analyzed using immunohistochemistry, Western blots and protein microarrays, only 49% could be validated^39^. Lack of cross-reactivity is best established when immunofluorescence is abolished by knockout of the target antigen, however we observed robust 596 immunostaining in ciliated cells from a patient homozygous for a rare Class I mutation that truncates CFTR upstream of the epitope, and similar results were obtained using antibodies 769 and 528. We identified the ciliary protein rootletin X1 and six other variants as candidate cross-reacting proteins because they have an amino acid sequence closely resembling the 596 epitope in CFTR. Super-resolution microscopy revealed bright clusters of 596 immunofluorescence arranged in circles around the bases of motile cilia where rootletin X1 is situated, although we note that the 596 immunofluorescence had a different appearance from that observed using anti-rootletin antibody, as expected for a large filamentous protein and distinct epitopes. Importantly, immunofluorescence was not detected in ciliated cells from all patients and its absence coincided with low rootletin expression according to immunoblots. This finding combined with the ability of 596 to immunostain heterologous rootletin X1 transfected into an epithelial cell line suggest it may be the cross-reacting protein detected by 596, 769 and 528 in ciliated cells. The conclusion that there is little, if any, CFTR protein in ciliated cells is congruent with previous results obtained by expressing CFTR in mouse airways using a promoter that is specific for ciliated cells^40^. Expression in ciliated cells did not correct the nasal bioelectric properties of CFTR knock-out mice measured in vivo, implying that ciliated cells are not normally the site of CFTR-dependent transport. The precise role of rootletin X1 remains poorly understood, although rootletin knock-out mice do have increased cilia breakage and lymphocyte infiltration into the lungs^34–37^. We found that 596- cells with little rootletin expression have shorter cilia and proliferate more slowly than 596 + cells, but whether these abnormalities have any clinical significance remains to be investigated.

Binding of 596, 769 and 528 to eGFP-rootletinX1 was shown in transfected parental CFBE41o^-^ cells that have very low levels of endogenous F508del CFTR mRNA. Moreover apical immunofluorescence in primary HBE cells was strongly correlated with the level of rootletin expression. Nevertheless we cannot exclude that there are other cross-reacting proteins, and this seems likely for 217 which binds a different epitope and did not immunostain heterologously expressed rootletinX1. We note that all antibodies tested for CFTR immunolocalization in well differentiated cells gave excellent colocalization with mCherry-WTCFTR in HBE cells cultured at the air–liquid interface, and they all detected CFTR specifically on immunoblots. Cross-reactivity was only a problem when immunostaining well differentiated (i.e. ciliated) cells for endogenous CFTR, and then only in cells from a subset (~ 70%) of cell donors.

We used ICCS to measure the degree of colocalization between particles with different fluorescence emission wavelengths^41^ rather than the more commonly used Pearson’s correlation coefficient because the latter is less accurate when each image channel has a different particle density^24^. Although ICCS overcomes this limitation, its accuracy also depends on several factors. Firstly, the Gaussian peak fitted to the cross-correlation function can become broad due to low intensity, spatially broad background signals in the images, especially when the signal-to-noise ratio is low and the signal from one or both detection channels is dim. We performed white noise correction by background subtraction to remove this contribution from the intensity means in the autocorrelation fits and cropped the correlation functions to limit the contribution of random correlations at higher spatial lags. Nevertheless, the low antibody signal observed in 596- cells made accurate estimation of correlated fractions challenging. Secondly, regions of interest had to be selected to be large enough to contain sufficient fluorescence fluctuations for analysis while avoiding spatial heterogeneity across the image region of interest^19^. ICCS provides an average description of the degree of colocalization of the target proteins rather than molecular (nm) scale information about their distributions. In the context of ICCS measurements, interacting means molecules that are part of a common complex within the focal spot, but does not imply direct molecular contact within the complex. Super-resolution microscopy provided insight into the distribution of fluorescence signals using different anti-CFTR antibodies and in cells from different donors (e.g. 596+ vs 596-). The absence of a distinct ring of clusters in 596- cells may result from lower protein expression or an altered distribution. Further studies are needed to understand the variable expression of rootletin between individuals and whether this influences mucociliary transport or basal cell proliferation after injury.

In summary, the results show that several CFTR antibodies can detect another protein in well differentiated HBE cells, probably a variant of the ciliary protein rootletin. They therefore suggest that ciliated cells are not a major site of CFTR protein expression. Cross-reactivity may not explain all reports of anomalous CFTR immunofluorescence however they encourage the validation of other CFTR antibodies using native tissues and well differentiated cultured cells homozygous that have Class I CFTR mutations.

## Supplementary Information

Supplementary Information.
