# Supplementary material for: Nonspecific binding of common anti-CFTR antibodies in ciliated cells of human airway epithelium
Source: Sci Rep. 2021 Dec 1;11:23256. doi: 10.1038/s41598-021-02420-x (PMC8636639; doi:10.1038/s41598-021-02420-x)
Supplement: Supplementary file 1 — Supplementary Information. [file 41598_2021_2420_MOESM1_ESM.pdf]

Antibody	Sequence	Domain
596[a]	WPSGGQMT	NBD2
528[a]	WPSGGQMT	NBD2
769[a]	WPSGGQMT	NBD2
217[a]	IYSRRLSQETGLE	R domain
450[a]	KRKNS-ILNPI	R domain
570[a]	PLERRLSLVPD-S	R domain
MM13-4[b]	RKGYRQRLELSD	N terminus

Supplementary Table S1. CFTR antibodies used in this study and reported locations of their epitopes. For details of epitope mapping see:

[a] https://cftrantibodies.web.unc.edu/sequence-alignment/

[b] https://www.sigmaaldrich.com/catalog/product/mm/05581?lang=en&region=CA

Supplementary Table S1

Patient	Age	Sex	FEV_1 (%)	Mutation	
F508-1	32	M	44	F508del/F508del	
F508-2	17	F	25	F508del/F508del	
F508-6	34	M	22	F508del/F508del	
F508-7	31	M	20	F508del/F508del	596+
F508-8	24	F	-	F508del/F508del	
F508-9	31	F	17	F508del/F508del	
F508-10	44	M	21	F508del/F508del	
CI-1	40	M	-	621+1 G>T/621+1 G>T	
F508-3	7	F	-	F508del/F508del	
F508-4	31	F	18	F508del/F508del	596 -
F508-5	30	F	32	F508del/F508del	
CI-2	22	M	22	1525-1G>A/1525-1G>A	

Supplementary Table S2. Characteristics of cell donors. Patient (de-identified), age, sex, lung function (% expected forced expiratory volume in 1 second, FEV_1) and CFTR genotype are shown. The apical aspect of ciliated cells cells from the first eight donors was clearly immunostained using CFTR antibody mAb596 (596+) whereas cells from the last four donors were not immunostained using mAB596 (596-).

Supplementary Table S2

Blast hits	Sequence
**596 antibody**	**WPSGGQMT**
**Rootletin isoforms X1-6**	WSPGGQML
**11 beta hydroxysteroid dehydrogenase 2**	WPSGG
**SIK3 isoform 1,2,3,4**	PSAGQM
**Glucocorticoid receptor**	SGGQM
**MRP6**	WPQGGQ
**utrophin**	PSGGQ
**LMTK1 isoform 1**	PSGGQ
**LMTK1 isoform 2**	PSGGQ
**FC receptor like protein 4**	PSGGQ
**PKD1**	WPSG
**Junctophilin 1**	WPSG
**Junctophilin 2**	WPSG

Supplementary Table S3. Results of BLAST search using the 596 epitope sequence WPSGGQMT on CFTR. A blocking peptide with this sequence abolished CFTR immunostaining.

Supplementary Table S3

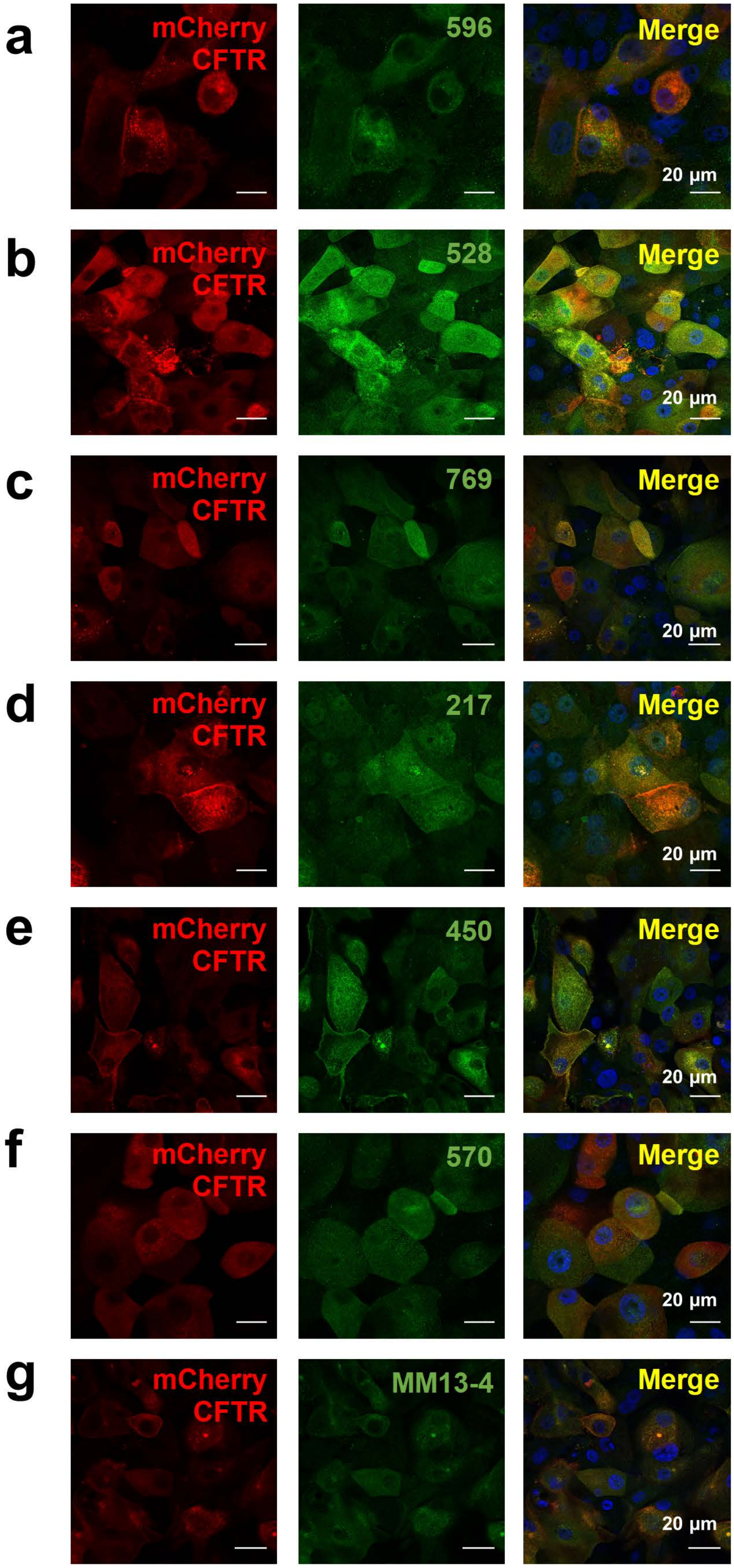

Supplementary Figure S1

Supplementary Figure S1. Confocal images of HBEs at the plane of the nucleus after transduction with adenovirus directing the expression of mCherry-WTCFTR. Cells were cultured at the ALI for 10-15 days and immunostained as described in the Materials and Methods.

Supplementary Figure S1 (cont.)

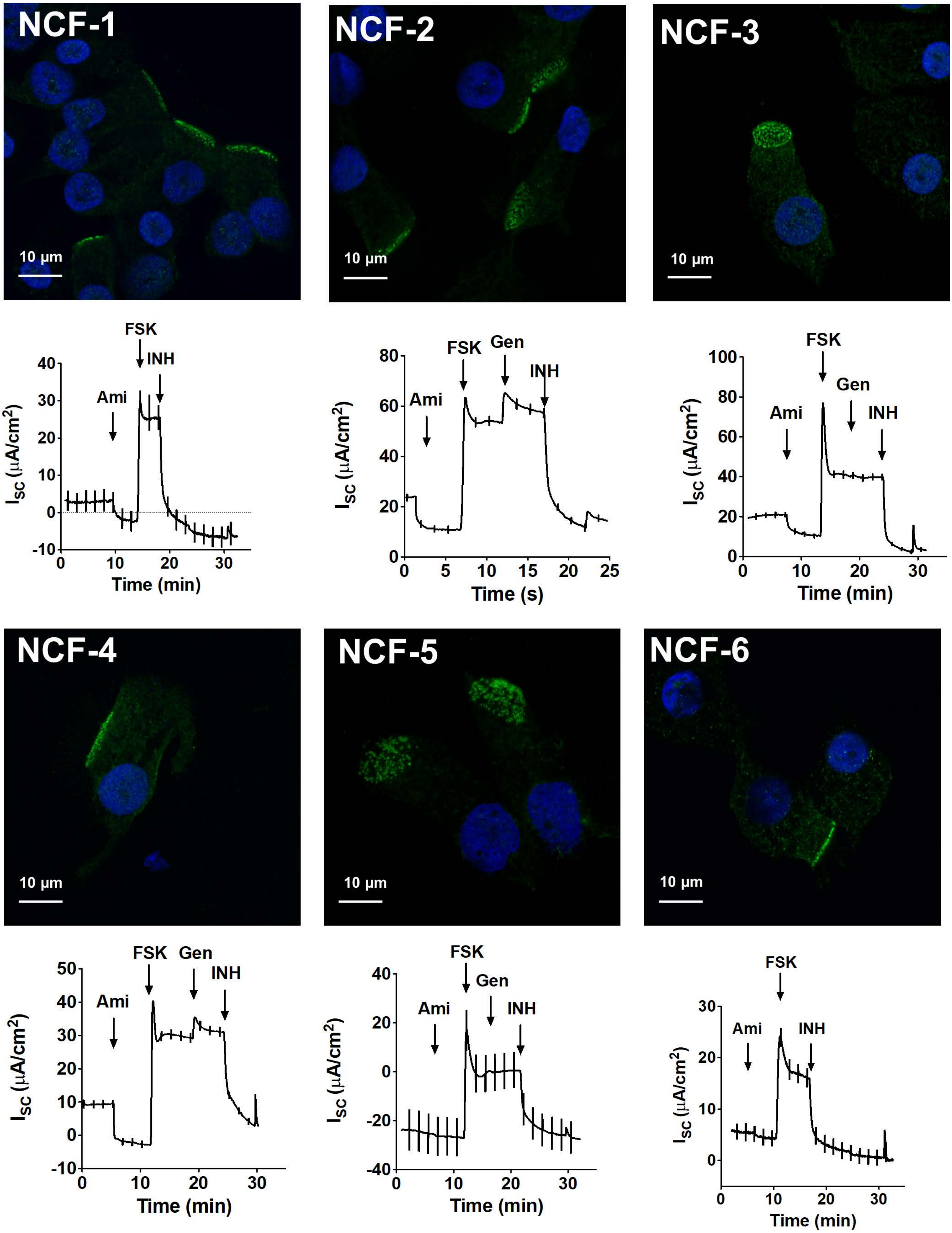

Supplementary Figure S2

Supplementary Figure S2. Apical 596 immunofluorescence and short-circuit current (I_{sc}) responses using cells from non-CF (NCF) donors. Representative 596 immunostaining images and CFTR functional responses obtained using well differentiated HBEs from six non-CF donors. Cells were treated with amiloride (Ami; 100 µM) to inhibit Na+ current. CFTR channels were activated using forskolin (FSK; 10 µM) and genistein (Gen; 50 µM) and inhibited by $CFTR_{inh}$-172 (INH; 10 µM).

Supplementary Figure S2 (cont.)

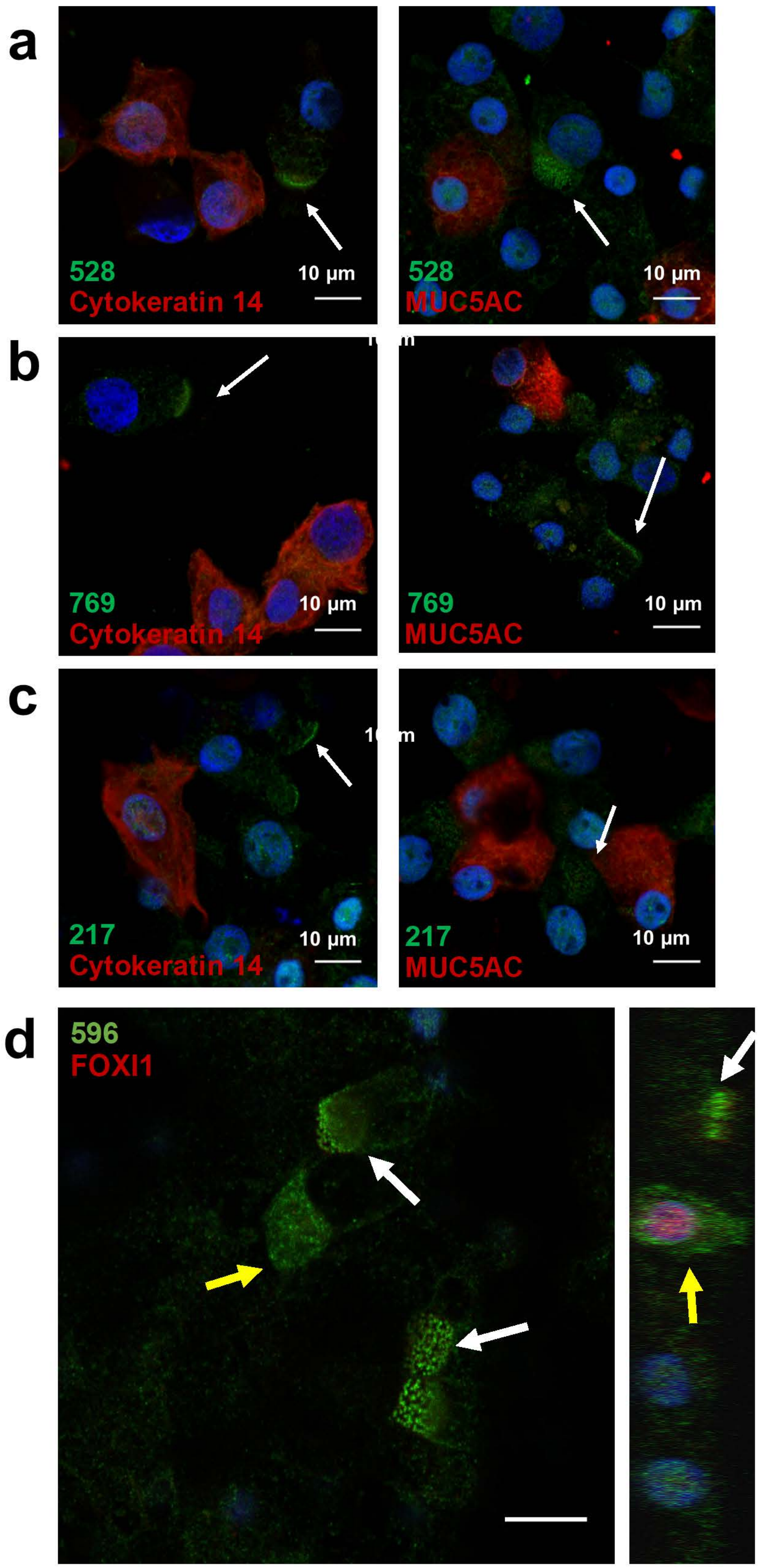

Supplementary Figure S3

Supplementary Figure S3. Apical immunostaining of ciliated cells by 528, 769, 217, and 596. (a-c) Non-CF cells co-immunostained using antibodies directed against the basal cell marker cytokeratin 14 (left) or goblet cell marker MUC5AC (right) in combination with CFTR antibodies (a) 528, (b) 769 or (c) 217. Note immunofluorescence is detected in ciliated cells but not basal or goblet cells. (d) Non-CF cells immunostained with 596 and antibody against the ionocyte marker FOXi1. Image represent a x-y view and y-z view. White arrow points to ciliated cells, yellow arrow points to ionocyte. Note similar 596 immunostaining for FOXi1-positive ionocyte and FOXi1-negative ciliated cells.

Supplementary Figure S3 (cont.)

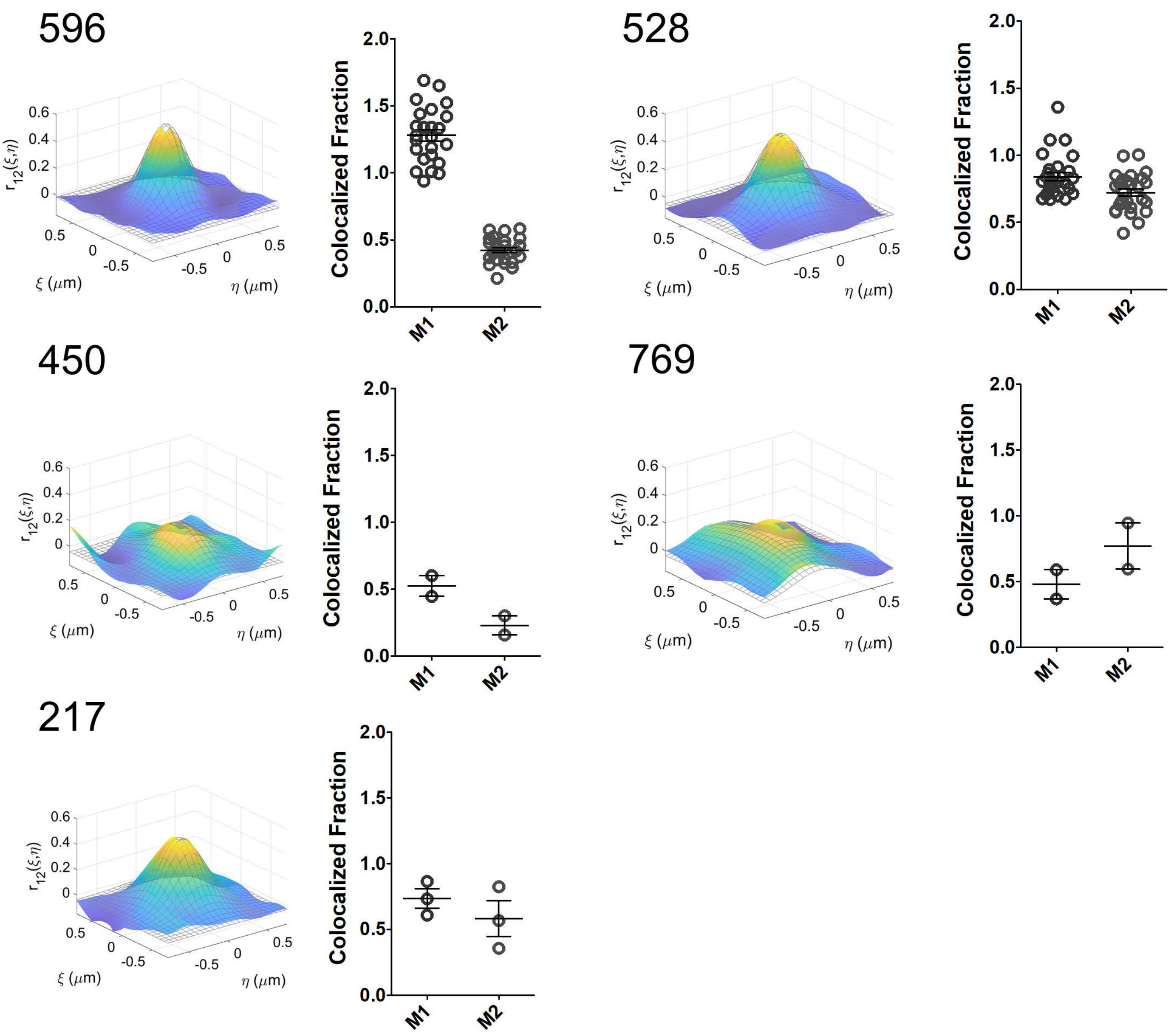

Supplementary Figure S4. Image cross-correlation spectroscopy (ICCS) analysis for cells co-immunostained using antibodies against the ciliary marker protein centrin-2 and CFTR. Representative ICCS analyses of centrin-2 immunofluorescence and that for each CFTR antibody used in the study. M1 and M2 are the fractions of the anti-CFTR and anti-centrin-2 signals that are colocalized, respectively. Values of M1>1.0 are likely due to noise in the measured immunofluorescence signals when the predicted colocalization is close to 100%.

Supplementary Figure S4

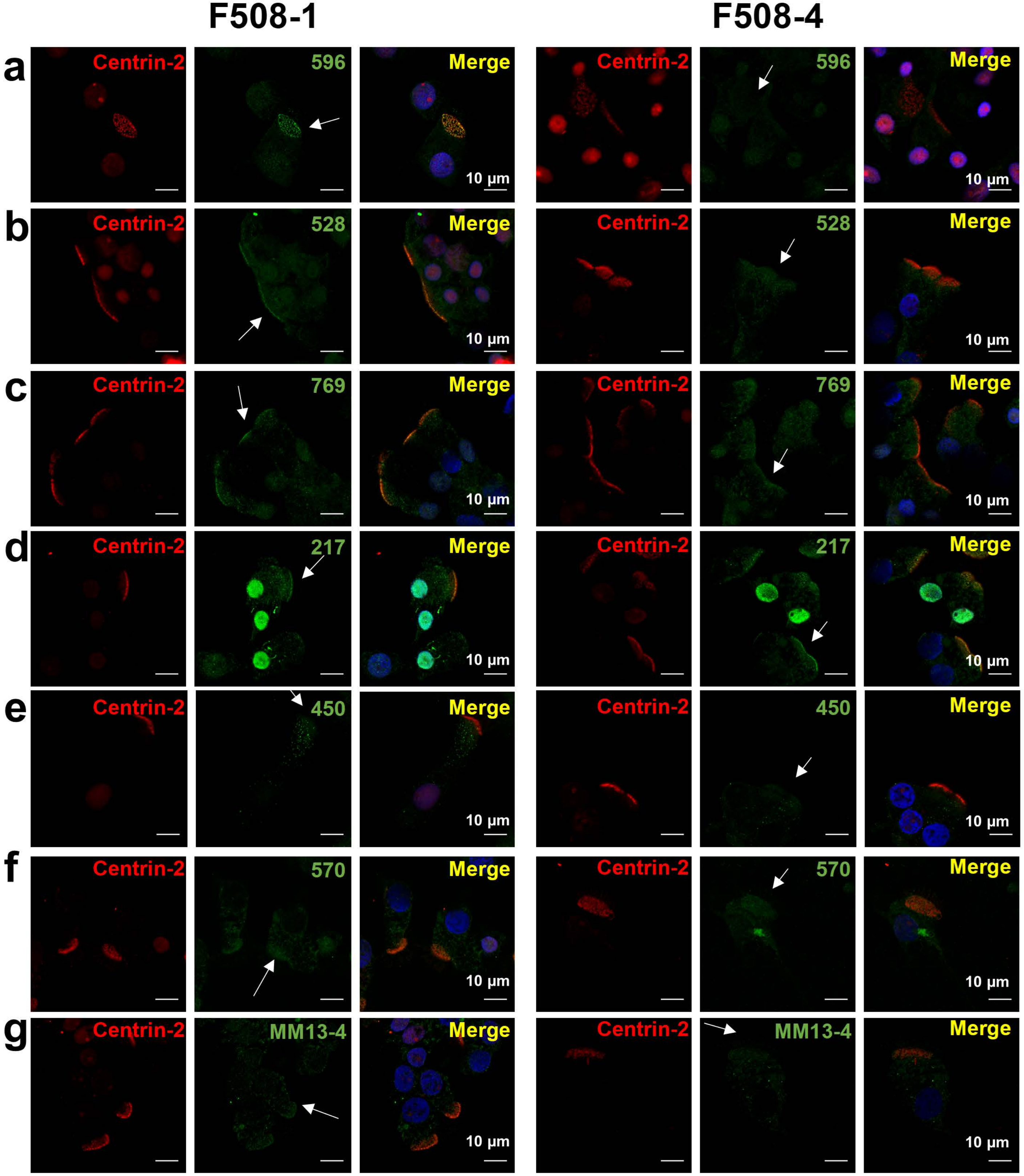

Supplementary Figure S5

Supplementary Figure S5. Ciliated F508del/F508del cells are immunostained by antibodies 596, 528, 769, and 217, but not by 450, 570 and MM13-4. Well differentiated HBE cells from two patients, one 596+ (left, F508-1) and the other 596- (right, F508-4) were co-immunostained using anti-centrin-2 (red, a marker of ciliated cells) and anti-CFTR antibodies (a) 596, (b) 528, (c) 769, (d) 217, (e) 450, (f) 570, and (g) MM13-4. White arrows indicate ciliated cells.

Supplementary Figure S5 (cont.)

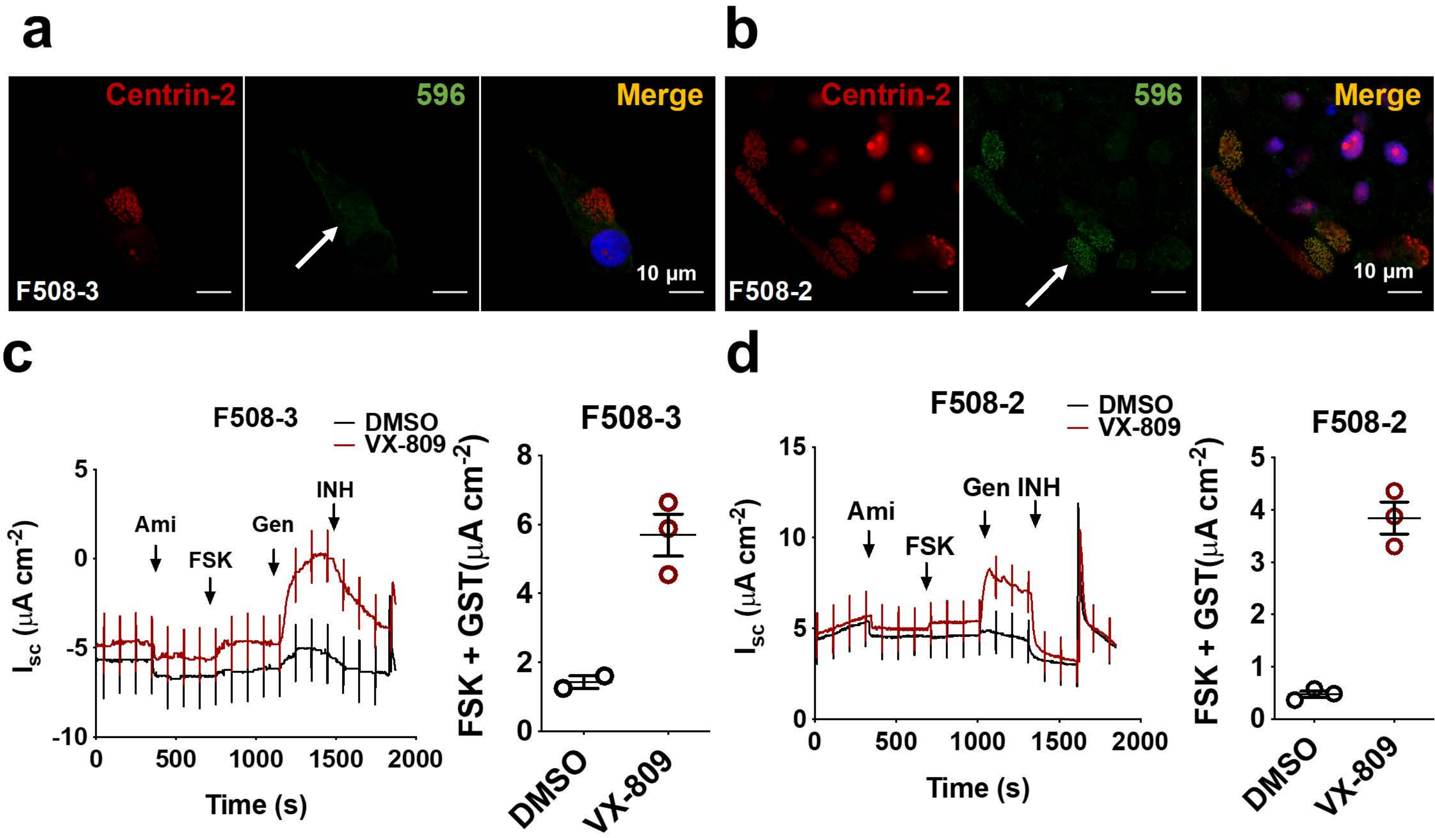

Supplementary Figure S6. Immunostaining of ciliated cells by 596 is not correlated with CFTR functional expression. Well differentiated F508del/F508del HBE cells were coimmunostained using anti-centrin-2 antibody (red) and 596 (green) and studied in parallel Ussing chamber experiments. (a,b) Examples of 596- (patient F508-3) and 596+ (patient F508-2) immunofluorescence. White arrows indicate ciliated cells in the 596 panels. (c,d) Short-circuit current recordings and summary histograms of 596- and 596+ cells after pretreatment with vehicle (DMSO, black) or the corrector VX-809 (1 µM, red) for 24 h. Sodium current was inhibited using amiloride (Ami; 100 µM). CFTR was activated using forskolin (FSK; 10 µM) and the potentiator genistein (Gen; 50 µM). Currents were inhibited by CFTRinh-172 (INH; 10 µM).

Supplementary Figure S6

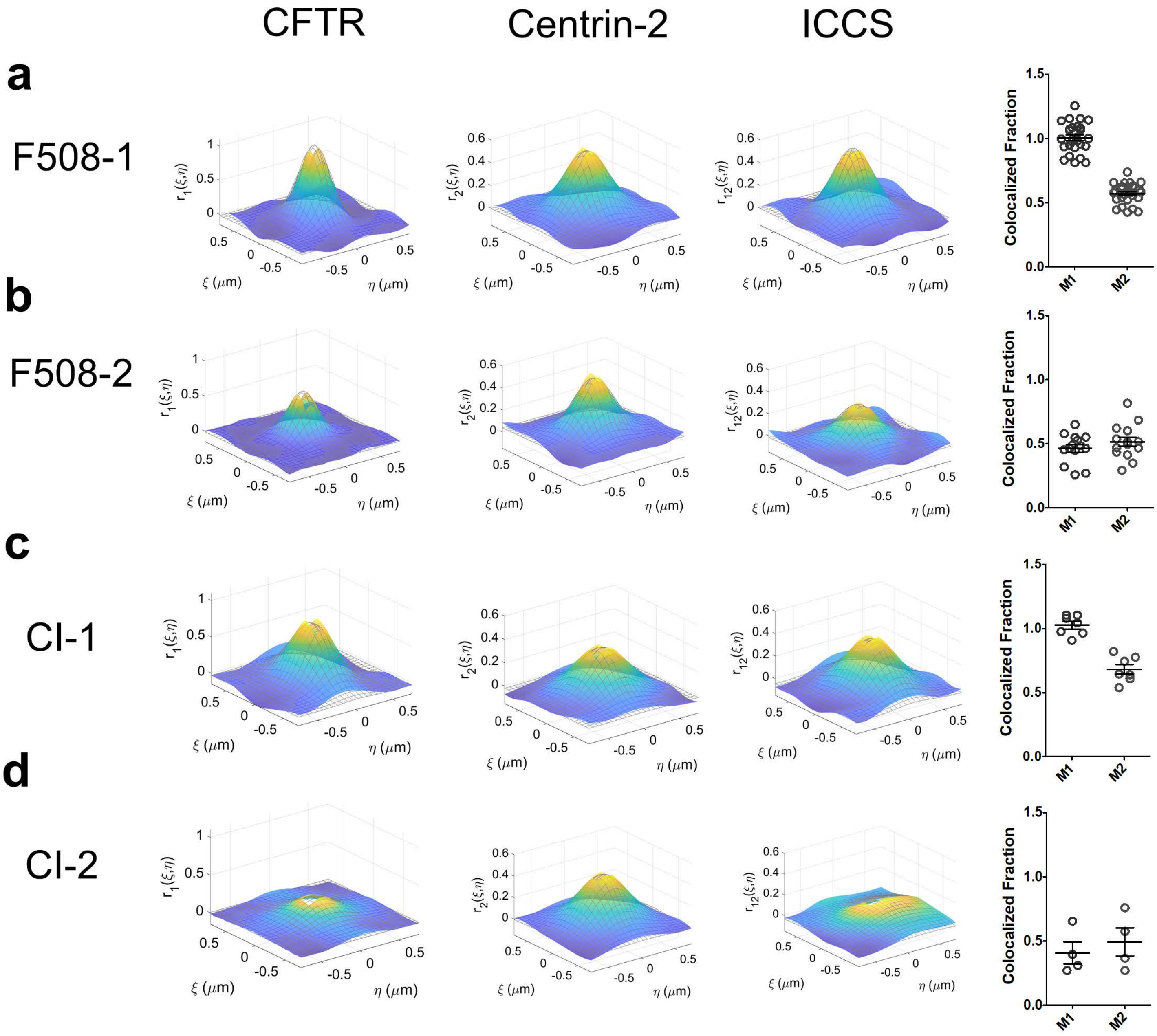

Supplementary Figure S7. Correlation functions and calculated co-localized fractions for 596 (M1) and centrin-2 (M2) antibody fluorescence using cells from different CF patients. (a) F508del/F508del cells (596+, from patient F508-1), (b) F508del/F508del cells (596-, from patient F508-2), (c) 621+1G>T/621+1G>T cells (596+, from patient CI-1) and (d) 1525-1G>A/1525-1G>A cells (596-, from patient CI-2).

Supplementary Figure S7

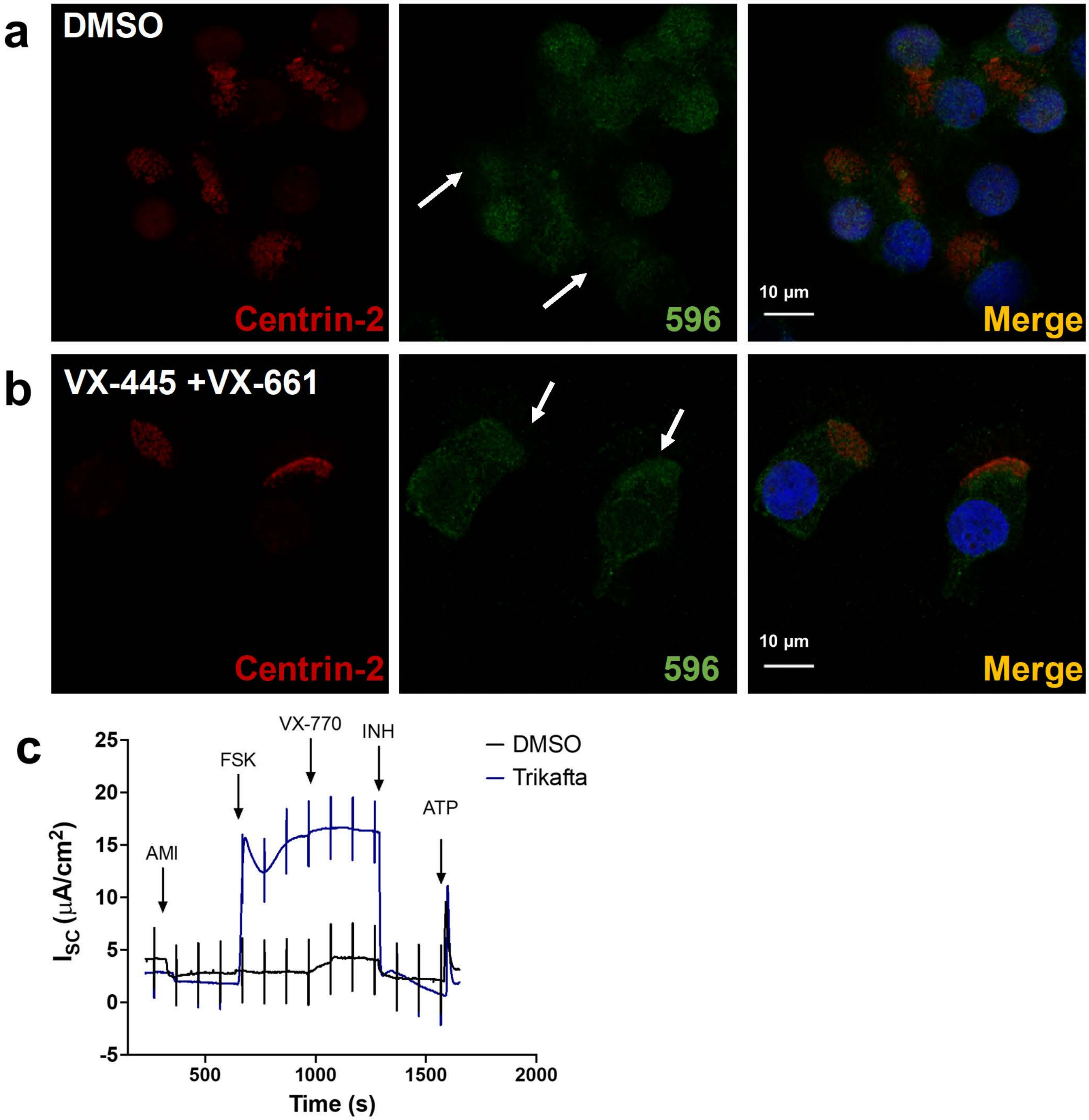

Supplementary Figure S8. Treatment with correctors VX-445 + VX-661 (elexacaftor + texacaftor) for 24 h does not increase apical staining in ciliated cells. Representative immunostaining of 596- cells from patient F508-3 using anti-centrin-2 (ciliated cell marker) and 596 antibodies after pretreating cells with (a) vehicle (0.1% DMSO) or (b) VX-445+VX-661 for 24 h. White arrows in the 596 images indicate ciliated cells. (c) Representative short-circuit recording of CF HBE cells from donor F508-3 after pretreatment with DMSO (black) or CFTR modulators used in Trikafta (VX-445;3 µM, VX-661; 3 µM, VX-770; 10 nM - Blue) for 24 h. AMI (amiloride), FSK (forskolin), VX-770 (ivacaftor), INH ($CFTR_{inh}$-172), ATP (purinoceptor agonist).

Supplementary Figure S8

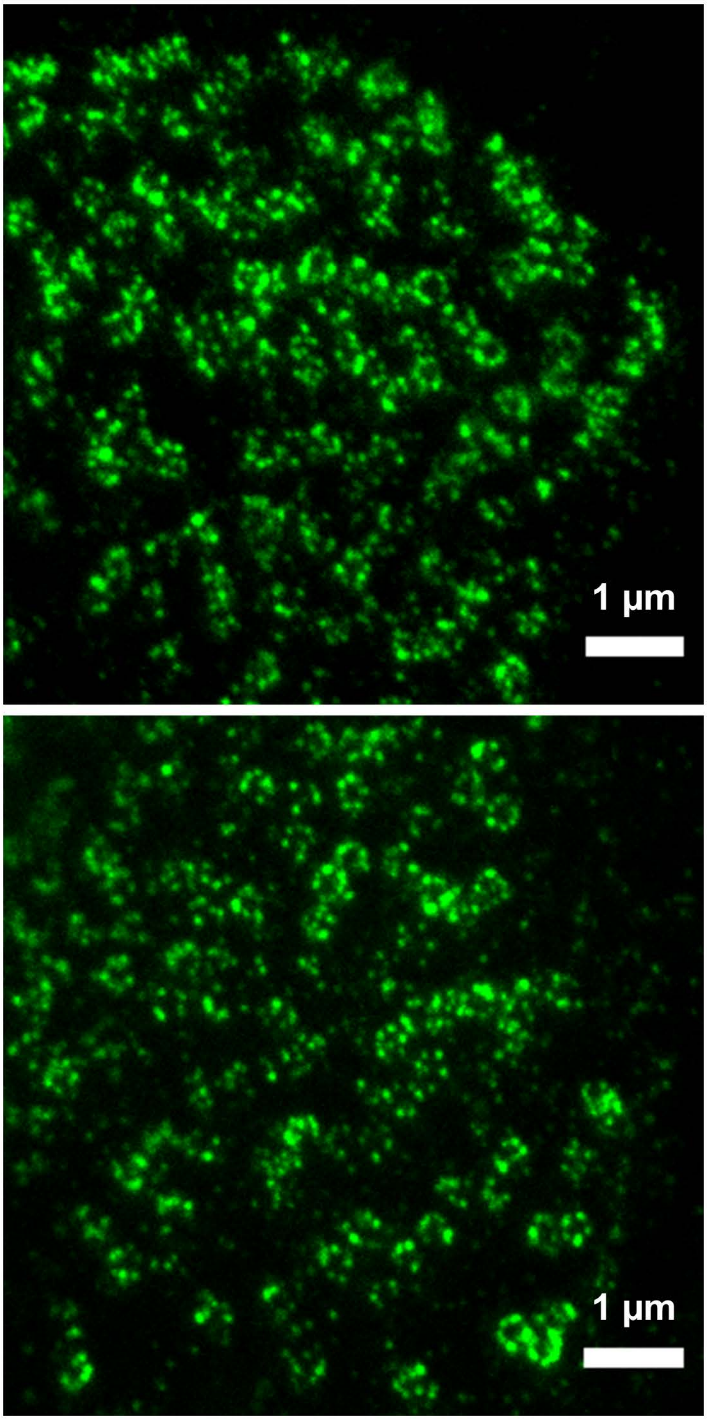

Supplementary Figure S9. Representative STED images of the apical pole of well-differentiated ciliated cells immunostained with 596 antibody. Cells used for both experiments are homozygous for the Class I mutation 621+1G>T/621+1G>T, which causes truncation of CFTR upstream of the 596 epitope.

Supplementary Figure S9

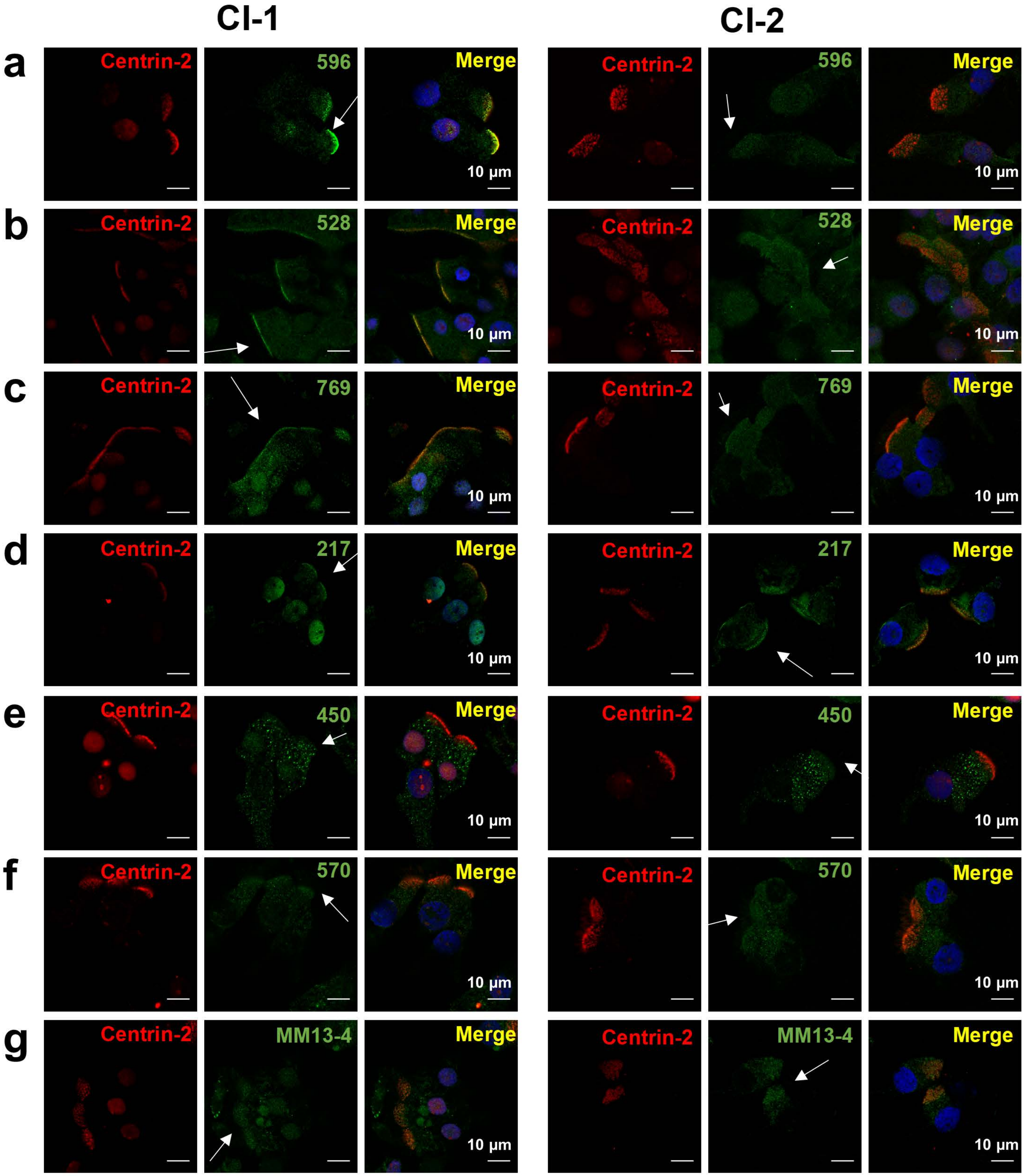

Supplementary Figure S10

Supplementary Figure S10. Ciliated Class I mutant cells that are immunostained by 596 are also detected using 528, 769, and 217, but not 450, 570 and MM13-4. Well differentiated HBE cells isolated from two patients, one 596+ (left, CI-1, 621+1G>T/621+1G>T) and the other 596- (right, CI-2, 1525-1G>A/1525-1G>A). Cells were co-immunostained using centrin-2 (red, marker of ciliated cells) and CFTR antibodies (a) 596, (b) 528, (c) 769, (d) 217, (e) 450, (f) 570, and (g) MM13-4.

Supplementary Figure S10 (cont.)

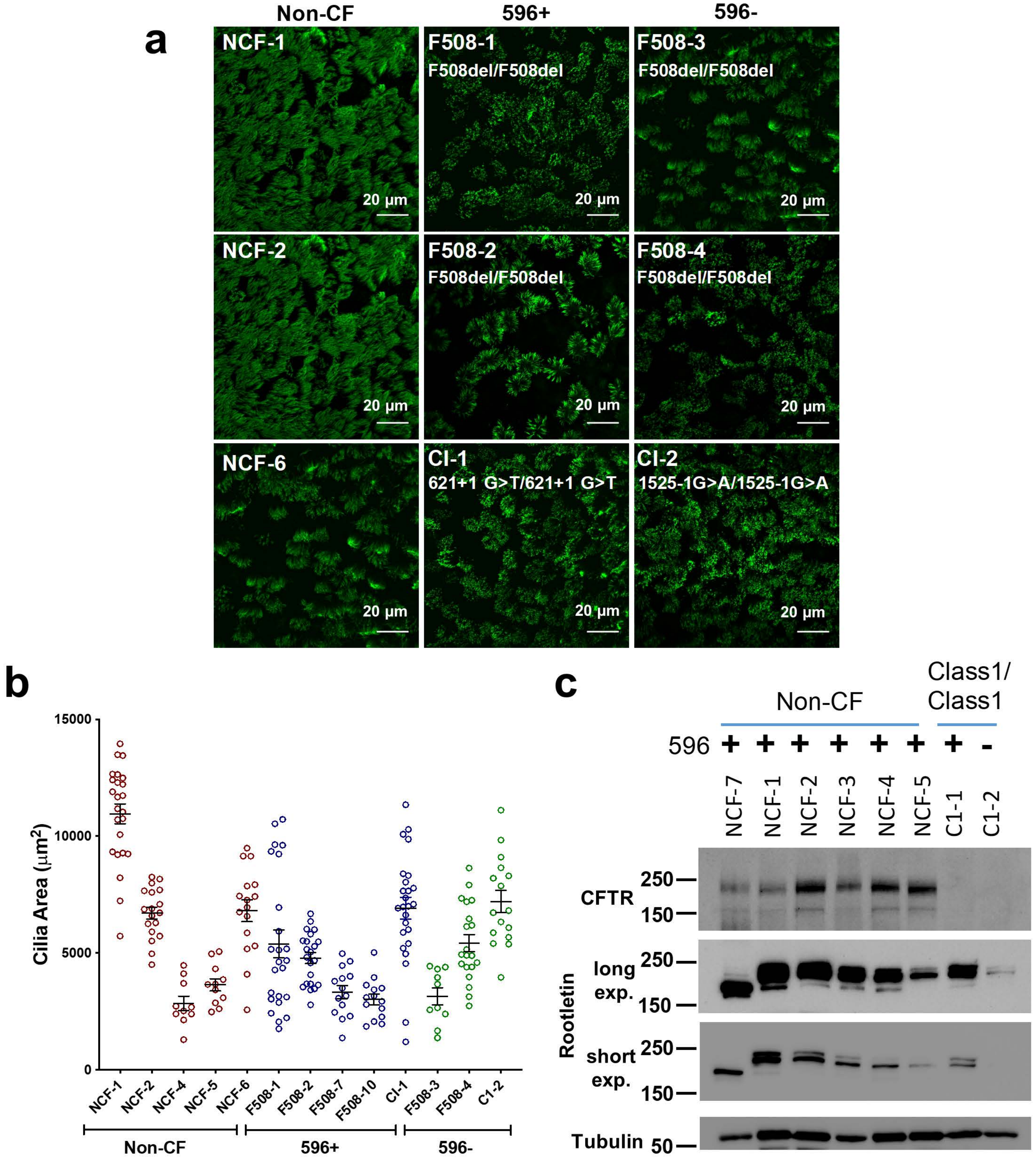

Supplementary Figure S11

Supplementary Figure S11. Ciliation varies but is not correlated with 596 immunostaining. (a) Tubulin immunostaining in non-CF, 596+ and 596- CF cells, including cells homozygous for F508del and a Class I mutation. (b) Area of ciliated cells in cultures from multiple patients in each category (mean ± SE, n = 10–98). Each point represents ciliated area from one field of view. (c) Immunoblot of lysates from 596+ and 596- HBE cells in panels A and B, probed with antibodies for CFTR, rootletin isoforms and tubulin. Note the absence of CFTR in Class I mutant cells and very low rootletin in 596- cells as shown using long and short exposures. Images taken from 2 immunoblots (top and bottom images show blot probed with 23c5 and anti-tubulin antibody. Middle two blots probed with anti-rootletin antibody after long and short exposures).

Supplementary Figure S11 (cont.)

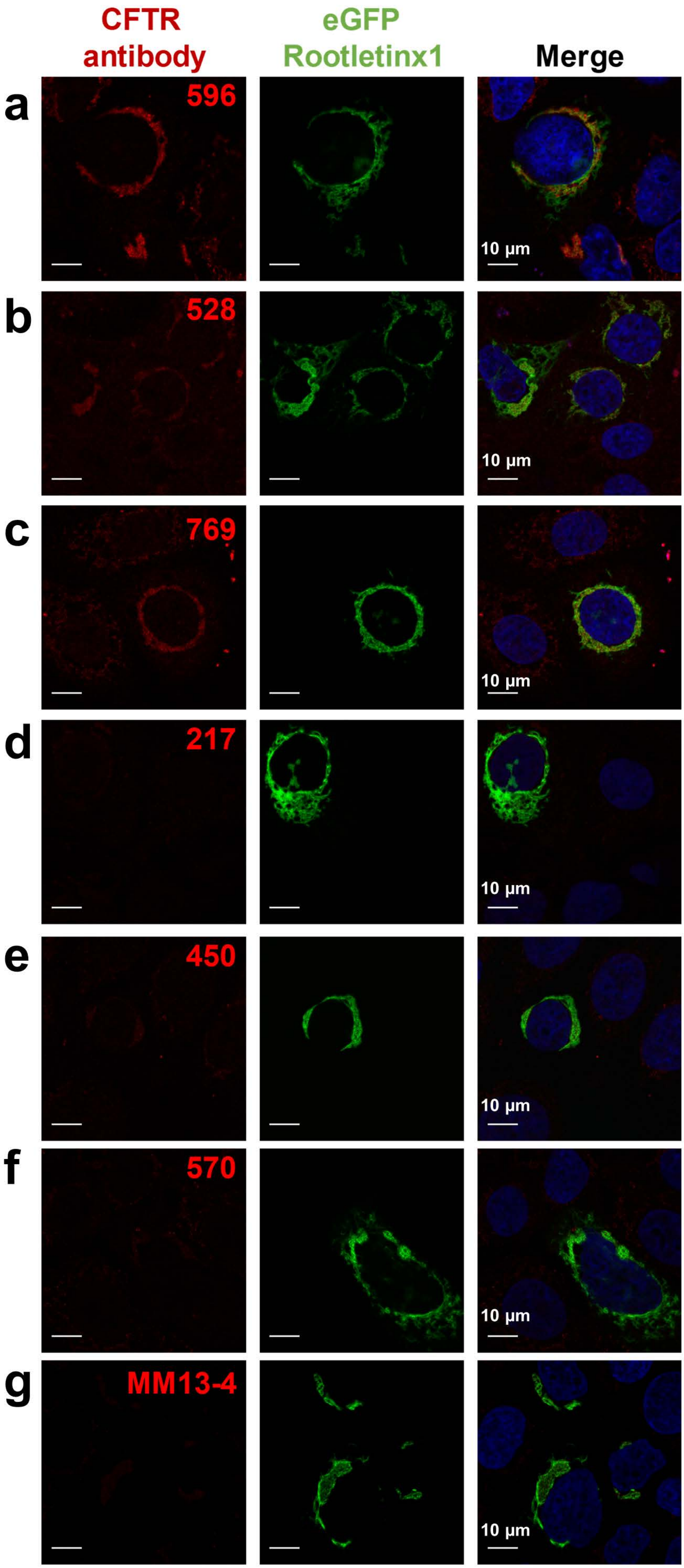

Supplementary Figure S12

Supplementary Figure S12. Heterologous rootletin X1 is detected by CFTR antibodies 596, 528 and 769, but not by 217, 450, 570. CFBE cells were transfected with eGFP-rootletin isoformX1 and immunostained using different CFTR antibodies: (a) 596, (b) 528, (c) 769, (d) 217, (e) 450, (f) 570 and (g) MM13-4.

Supplementary Figure S12 (cont.)

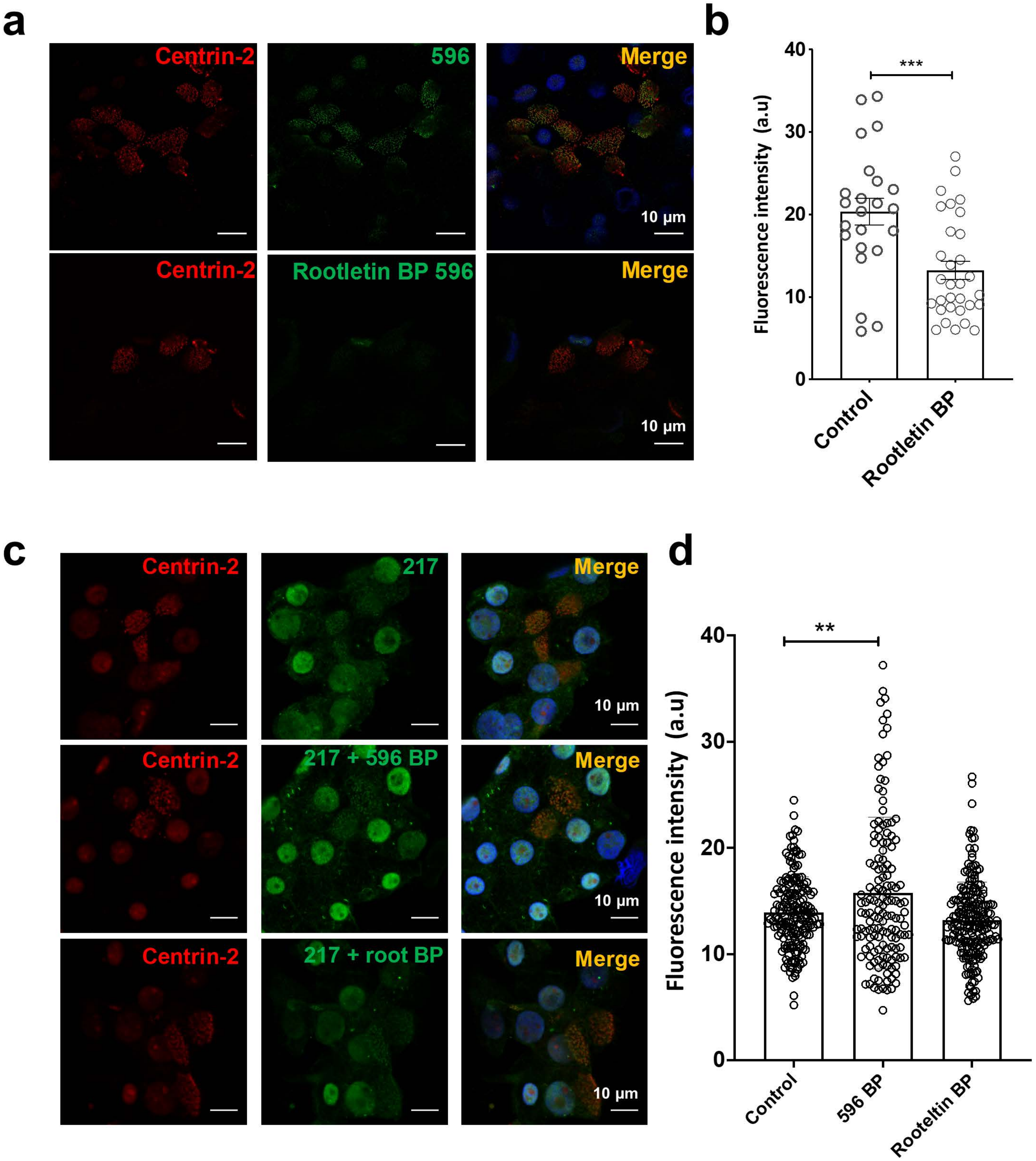

Supplementary Figure S13

Supplementary Figure S13. Blocking peptide with rootletinX1 sequence inhibits 596 staining at the bases of cilia. (a) Representative confocal images of 596 staining of differentiated pHBE cells without (top) and with rootletin blocking peptide (bottom). (b) Average intensity of apical immunofluorescence signals detected with 596 immunostaining in the presence and absence of blocking peptide with the rootletin sequence (***p=0.0004). Confocal imaging was performed using a different imaging system therefore intensities were not normalized to other fluorescence intensity comparisons. (c) Representative confocal images of 450 staining of differentiated pHBE cells without (top) and with rootletin blocking peptide (bottom). (d) Average intensity of apical immunofluorescence signals detected with 450 immunostaining in the presence and absence of blocking peptide with the rootletin sequence. Confocal imaging was performed using a different imaging system therefore intensities were not normalized to other fluorescence intensities.

Supplementary Figure S13 (cont.)
